# Examining the Impact of Socioeconomic Position Across the Life Course on Cognitive Function and Brain Structure in Healthy Aging

**DOI:** 10.1093/gerona/glad068

**Published:** 2023-02-23

**Authors:** Céline De Looze, Naiara Demnitz, Silvin Knight, Daniel Carey, Jim Meaney, Rose Anne Kenny, Cathal McCrory

**Affiliations:** The Irish Longitudinal Study on Ageing (TILDA), School of Medicine, Trinity College Dublin, Dublin, Ireland; Global Brain Health Institute, Trinity College Dublin, Dublin, Ireland; Danish Research Centre for Magnetic Resonance, Centre for Functional and Diagnostic Imaging and Research, Copenhagen University Hospital—Amager and Hvidovre, Hvidovre, Denmark; The Irish Longitudinal Study on Ageing (TILDA), School of Medicine, Trinity College Dublin, Dublin, Ireland; The Irish Longitudinal Study on Ageing (TILDA), School of Medicine, Trinity College Dublin, Dublin, Ireland; The National Centre for Advanced Medical Imaging (CAMI), St. James’s Hospital, Dublin, Ireland; The Irish Longitudinal Study on Ageing (TILDA), School of Medicine, Trinity College Dublin, Dublin, Ireland; Mercer’s Institute for Successful Ageing (MISA), St. James’s Hospital, Dublin, Ireland; The Irish Longitudinal Study on Ageing (TILDA), School of Medicine, Trinity College Dublin, Dublin, Ireland

**Keywords:** Brain volumes, Cognitive status, Diagonal reference model, Social mobility

## Abstract

This study explores the relationship of life-course intergenerational social mobility with cognitive function and brain structure in older adults using Diagonal Reference Models. Data from the Irish Longitudinal Study on Ageing, a population-based cohort of adults aged 50 years and older (*N* = 4 620 participants; mean age: 66.1; standard deviation: 9.1; 55% female) was used for analysis. Brain magnetic resonance imaging data were available for 464 participants. Social mobility was characterized as the difference between childhood socioeconomic position (SEP; ie, father’s occupation) and adulthood SEP (ie, own occupation). The Montreal Cognitive Assessment (MoCA), the Mini-Mental State Examination (MMSE), cortical thickness, and total gray matter volume (GMV) served as global cognitive and brain measures. Exploratory analyses included the volumes of the ventromedial prefrontal cortex (vmPFC), anterior cingulate (AC), hippocampus, and amygdala. A social gradient in cognitive function was observed among the intergenerationally stable; brain structure was not as clearly socially patterned. Adulthood SEP was significantly associated with MoCA (weight = 0.76; *p* < .001), MMSE (weight = 0.91; *p* < .001), GMV (weight = 0.77; *p* = .002), and AC volume (weight = 0.76; *p* < .001), whereas childhood SEP was associated with vmPFC volume (weight = 1.00; *p* = .003). There was no independent association of social mobility with any of the outcomes. Together our results suggest that both childhood and adulthood SEP are important in shaping later-life brain health, but that adulthood SEP predominates in terms of its influence. This is potentially an important insight as it suggests that brain health may be modifiable if socioeconomic circumstances change.

Socioeconomic position (SEP), usually operationalized using class or education, is one of the strongest determinants of brain health over the life course (1), and many population-based studies have documented social gradients in cognitive aging and dementia risk at older ages (2,3). The ubiquity and magnitude of the association between markers of SEP and brain health have prompted a burgeoning interest in the pathways through which socioeconomic factors exert their influence as a modifiable risk factor for dementia. According to a recent paper published in the *Lancet* (4), less education in early life was associated with a population-attributable fraction of 7.1% for dementia risk. However, many of the other modifiable downstream risk factors for dementia identified in that review (midlife hypertension, obesity, hearing loss, smoking, depression, physical inactivity, social isolation, diabetes, alcohol consumption, traumatic brain injury, and air pollution) are strongly socioeconomically patterned (5), suggesting that the impact of intervening on education may, in fact, be much larger.

Although a number of studies have documented a positive association between SEP and cognition (6–8) and reduced dementia risk (9,10), fewer studies have examined the relationship between SEP and brain structure in old age. Using magnetic resonance imaging (MRI)-derived measures, differences in brain structure can be observed years prior to a dementia diagnosis (11), and can therefore be a more sensitive marker of life-time environmental effects on brain health. For instance, lower SEP has previously been associated with reduced cortical thickness in middle-aged adults (12) and reduced total gray matter volume (GMV) across the life span (13). Although no consensus has yet been reached regarding the topographic pattern of these effects, reduced GMVs in the hippocampus, anterior cingulated (AC) cortex, and the prefrontal cortex have previously been associated with SEP (14).

These neuroanatomical correlates of SEP may reflect regions particularly susceptible to environmental differences, such as enriched or impoverished environments and early-life stressors. Low childhood SEP has been linked with compromised neurodevelopment in early life (6,15,16), including smaller GMVs, slower growth of the hippocampus, and impaired development in subcortical structures of the brain related to emotion processing such as the amygdala, thalamus, and striatum. However, as studies have typically not examined the role of SEP on the aging brain, it remains to be convincingly demonstrated that these early emerging deficits are preserved into older adulthood (6). For instance, a recent Swiss study reported legacy effects of high childhood SEP with structural brain properties including higher GMVs and myelin content that survived adjustment for adulthood SEP (17).

Three main life-course models may be advanced to explain the effects of SEP on brain health. The *critical period* (18) model suggests that there are developmental periods during which the effects of SEP on health are particularly salient, and that adverse exposures occurring during these critical windows cause permanent and irreversible alterations to brain structure or function that compromise longer-term cognitive functioning. The *accumulation* model by contrast holds that cumulative exposure to socioeconomic adversity across the life course erodes health in a dose-dependent manner. Within this framework, childhood SEP represents a risk factor for dementia because it is such a powerful predictor of continuing socioeconomic dis/advantage into adulthood and later life. These risks can be either single recurrent (eg, ongoing poverty) or additive (eg, poverty → low education → lower occupational position → lower job complexity) with repeated “hits” having a compounding effect in terms of their influence on cognitive health over a long span of years (19). Indeed, many studies have reported that adulthood SEP either partially (20) or fully (21) mediates the association of childhood SEP with cognitive performance in later life.

Although these first 2 models consider the impact of timing and duration of exposure, a third model, that is, *social mobility*, explores the extent to which intra- or intergenerational transitions in socioeconomic circumstances over the life course modify risk for dementia. Examining the cognitive function of individuals who are socially mobile represents an important test of these different life-course models. If those who are socially mobile have similar risk profiles to those who are nonmobile at lower SEPs, this supports the scarring effects of the early childhood social environment. If social mobility ameliorates the risk for dementia in later life, this suggests a degree of plasticity and prompts a search for the SEP-related mechanisms through which mobility confers its protective effect.

Exploring mobility effects is usually accomplished by the cross-classification of childhood and adulthood SEP to create a contingency table of different socioeconomic trajectories: *stable high* (high childhood and adulthood SEP), *stable low* (low childhood and adulthood SEP), *downwardly mobile* (high childhood, low adulthood SEP), and *upwardly mobile* (low childhood, high adulthood SEP). However, this common approach does not in itself constitute a strong test of whether social mobility modulates the risk of cognitive impairment in later life. Being directly calculated from childhood and adulthood SEP, social mobility is linearly dependent on these parameters, and therefore, its effect cannot be disentangled from childhood and adulthood effects (22). Diagonal reference models (DRMs) by contrast can determine social mobility effects over and above the contribution of childhood and adulthood SEP. The guiding assumption of DRMs is that socially stable individuals represent the most suitable point of reference, reflecting the true characteristics of the given class. The effect of being mobile is modeled as a weighted sum of the diagonal effects (as these were the 2 classes they were socialized by) and 2 weight parameters (bounded by the values of 0–1), reflecting resemblance to the childhood and adulthood class. A high value of the childhood weight means that those who are mobile resemble the nonmobile in their childhood class, whereas a high weight for adulthood means those who are mobile resemble more the adulthood class they join. If the weight is 0.5, it means childhood and adulthood are equally influential. The effect of mobility is then estimated as the *independent* effect of the change in SEP after accounting for the relative importance of childhood and adulthood SEP.

In this paper, using DRMs, we examine the independent effect of social mobility on 2 established measures of global cognitive function among a large sample of community-dwelling older adults. We innovate further by applying the same methodological approach to structural brain data for a subset of participants who completed an MRI scan, allowing to explore whether life-course SEP is associated with measures of brain structure in late adulthood.

## Method

### Sample

The Irish Longitudinal Study on Ageing (TILDA) is a large population-based study of a nationally representative sample of community-dwelling older adults aged 50 years and over, resident in the Republic of Ireland. TILDA’s random sampling procedure and study design have been described elsewhere (23). Briefly, participants have been followed biennially from Wave 1 (data collection baseline: 2009–11; *N* = 8 504) and this analysis includes data from Wave 3 (2014–15). Socioeconomic and cognitive data were available for 4 620 participants. A subset (*N* = 464) also had brain measures for complete case analyses (see Supplementary Figure 1 for a detailed breakdown). Ethical approval was granted by the Trinity College Faculty of Health Sciences Research Ethics Committee, Dublin, Ireland. Protocols conformed with the 1964 Declaration of Helsinki and its later amendments. Signed informed consent was obtained from all respondents prior to participation. Additional ethics approval was received for the MRI substudy from St. James’s Hospital/Adelaide and Meath Hospital, Inc., National Children’s Hospital, Tallaght (SJH/AMNCH) Research Ethic Committee, Dublin, Ireland. Those attending for MRI were also required to complete an additional MRI-specific consent form.

### Socioeconomic Position and Social Mobility

Childhood SEP was drawn from retrospective data on father’s occupation (24) when the participant was aged 14. Adulthood SEP was estimated using the participant’s current occupation or highest paid job if retired. Occupations for both childhood and adulthood were coded following the Irish Central Statistics Office social class schema: (a) professional/ managerial, (b) nonmanual, (c) skilled manual/semi-skilled, (d) unskilled, and (e) never worked. Due to small cell sizes, occupational categories were collapsed into 3 groups: (a) professional/managerial, (b) nonmanual/ skilled manual, and (c) semi-skilled/unskilled/never worked.

### Global Cognitive Function

The Montreal Cognitive Assessment (MoCA) (25) and the Mini-Mental State Examination (MMSE) (26) were used to assess cognitive impairment. The MoCA is a well-established 30-point test that is used to screen for mild cognitive deficits and is commonly applied to cognitively intact older adults. The 30-point MMSE test is commonly used for dementia screening. For both tests, a binary variable was generated to define cognitive impairment using a cutoff point of <24 on the MoCA (27,28) and a cutoff point of <26 on the MMSE (29).

### Brain Structure

MRI scanning was completed at the National Centre for Advanced Medical Imaging, St. James’ Hospital, Dublin, via 3T Philip’s Achieva system and 32-channel head coil. The protocol included a variety of scans, including a T1-weighted magnetic resonance image acquired using a 3D Magnetization Prepared Rapid Gradient Echo (MP-RAGE) sequence, with the following parameters: field of view (mm): 240 × 218 × 162; 0.9 mm isotropic resolution; SENSE factor: 2; repetition time: 6.7 ms; echo time: 3.1 ms; flip angle: 9. All T1-weighted images were analyzed using the FreeSurfer software version 6.0 (30). All unprocessed input volumes were inspected for evidence of image artifact. Total and regional GMVs as well as cortical thickness were measured automatically via FreeSurfer pipeline, and cortical and subcortical segmentations were manually inspected using Freeview. Total vmPFC, AC (rostral and caudal), and hippocampal and amygdala volumes were generated using the sum of their left and right respective volumes. These regions of interest are strongly implicated in cognitive aging and strongly influenced by stress (31). Estimated total intracranial volume (eTIV) was also extracted for head-size adjustment in statistical analyses.

### Covariates

We controlled for age (years), sex, and childhood health (self-reported as Excellent [= 1] to Poor [= 5]; as well as eTIV when the outcomes were brain volumes) as potential confounders of the relationships between SEP, cognitive function, and brain structure. We adjusted for age and sex as patterns of intergenerational mobility, and cognitive function may vary across these demographic characteristics. We also adjusted for retrospectively reported childhood health as it can affect selection into adulthood SEP as well as cognitive functioning in later life. It is important to adjust for eTIV as it varies by age and sex, and is associated with brain volumetric measures. Additional variables, envisaged as potential mediators including pre-existing self-reported physician-diagnosed cardiovascular diseases and events (CVDEs), included history of angina, heart attack, congestive heart failure, stroke, transient ischemic attack, atrial fibrillation, and diabetes. Data were pooled to create a dichotomous CVDE measure, for the absence (CVDE free) or presence (≥1) of CVDEs. Self-reported chronic conditions included lung disease, asthma, arthritis, osteoporosis, cancer, stomach ulcer, varicose ulcer, liver disease, thyroid disease, or kidney disease. Participants were classified as having no chronic condition or chronic conditions ≥1. Average-seated systolic and diastolic blood pressure were measured using an Omron blood pressure monitor (2 readings 1 minute apart). Waist–hip ratio was estimated by dividing waist and hip measurements from each participant. Waist and hip circumferences were recorded to the nearest 0.01 m using a flexible tape measure (Seca Ltd, Birmingham, UK). Smoker status was defined as Never, Past, or Current. The CAGE questionnaire (32) was used as a measure of problematic alcohol consumption and is represented as a binary variable. Physical activity was recorded through the International Physical Activity Questionnaire (IPAQ (33); short-form) and is represented as a 3-level variable (low/moderate/high). Depressive symptoms were assessed using the Centre for Epidemiological Studies—Depression scale (CES-D (34)) and are represented as a continuous variable. Medications were classified using the Anatomical Therapeutic Chemical (ATC) classification codes questionnaire. Antihypertensive medication included ATC codes C02 (antiadrenergic agents), C03 (diuretics), C07 (β blockers), C08 (calcium-channel blockers), and C09 (angiotensin-converting enzyme inhibitors). Antidepressant medication was classified by ATC code N06A. Dichotomous variables were generated to indicate the usage of cardiovascular and antidepressant medications, respectively. Premorbid intelligence was measured using the National Adult Reading Test (NART) (35). The NART error score was generated using the Beardsall & Brayne algorithm (36). The lower the error score, the higher premorbid intelligence.

### Statistical Analyses

DRMs (22) were applied to examine the effect of intergenerational social mobility on cognitive function and brain structure. The MoCA and MMSE binary variables (1 = impairment vs 0 = no impairment) served as cognitive outcomes. Brain structure outcomes (continuous) included total GMV and cortical thickness, and the volumes of the vmPFC, AC, hippocampus, and amygdala. DRMs provide estimates for the socially stable and the socially mobile groups separately. The class coefficients for the socially stable groups indicate the class-specific deviations from the constant. The estimated intercepts for the socially mobile are modeled as the weighted sum of the diagonal effects (ie, those who are intergenerationally stable in their social class position) and 2 weight parameters quantifying the relative importance of childhood and adulthood SEP. To test for independent mobility effects, a series of dummy variables were created to represent mobility trajectories (from childhood SEP to adulthood SEP): (a) in any direction, (b) upward, and (c) downward. Baseline models included as predictors childhood SEP and adulthood SEP and the set of control variables which were mean centered with respect to age, childhood health, and eTIV. In subsequent models, mobility in any direction, upward mobility and downward mobility, was added independently as dummy variables. The fits of these nested models were compared with the baseline model using the Akaike and Bayesian Information Criteria (AIC and BIC). Analyses were conducted in Stata V.15 (StataCorp LLC, College Station, TX) (37). The DRM models were implemented using the “drm” module developed by Kaiser (38).

As a sensitivity check, the DRM analyses were repeated while controlling additionally for cardiovascular diseases and events, chronic diseases, blood pressure, waist–hip ratio, smoking status, alcohol consumption, physical activity, depression, antidepressants and antihypertensive medications (second set of models), and premorbid verbal intelligence as well (third set of models). Effect modifications were tested by fitting a sex × SEP interaction term in baseline models and models testing for independent mobility effects.

Finally, to enable comparisons with prior literature, we also estimated a set of conventional regression models to examine the effect of social mobility on cognitive function and brain structure. Social mobility status was characterized using the cross-classification of childhood and adulthood SEP into 5 groups: (1) stable professional/ managerial, (2) stable nonmanual/skilled manual, (3) stable semi-skilled/unskilled/never worked, (4) upwardly mobile, and (5) downwardly mobile. The dependent variables were modeled using binary logistic regression with respect to MoCA and MMSE, and ordinary least squares regression with respect to the structural brain measures. The stable professional/managerial class represented the reference group as they enjoyed high SEP in childhood and adulthood. As per the baseline DRMs, we controlled for age, sex and childhood health, and adjusted additionally for eTIV when the outcome was a brain volumetric measure.

## Results

### Data Descriptives

The characteristics of the main study sample are described in Table 1 (*N* = 4 620). Mean age was 66.1 years (*SD* = 9.1) and 55.6% were female. Mobility was common, with 38% of the participants upwardly mobile and 18% downwardly mobile. The stable professional group represented 11.5% of participants; the stable nonmanual/skilled manual 22%, and the stable semi-skilled/unskilled/never worked 10.5%. Compared to the stable professional group, the stable semi-skilled/unskilled/never worked group was older (*p* = .03) and included a higher proportion of women (*p* < .001). They were more likely to report poorer childhood health (*p* < .001), to have cardiovascular (*p* < .001) and chronic diseases (*p* = .01). They were more likely to report poorer lifestyle habits and higher levels of depressive symptoms and were more likely to take antihypertensive and antidepressant medications (*p* < .001). The health characteristics of the upwardly and downwardly mobile groups ranked intermediate between the stable groups, suggesting shared characteristics with childhood and adulthood SEP. The sample characteristics for the MRI sample (*N* = 464) are given in Supplementary Table 1.

**Table 1.** Descriptive Characteristics of the Study Sample (*N* = 4 620), Per Social Mobility Category Compared to the Excluded Participants

	Study Sample (*N* = 4 620)	Upwardly Mobile (*N* = 1 750)	Stable Professional (*N* = 534)	Stable Nonmanual/Skilled Manual (*N* = 1 022)	Stable Unskilled/Never Worked (*N* = 487)	Downwardly Mobile (*N* = 827)	Excluded (*N*=1 943)
Age (mean, *SD*)	66.1 (9.1)	66.1 (8.8)	65.9 (9.3)	65.6 (9.1)	67.1 (9.5)*	65.8 (9.2)	67.2 (10.1)***
Gender: Female (%)	55.6	51.2	55.1	52.7	64.3***	64.2**	56.2
Childhood health: Fair/Poor (%)	5.9	5.8***	5.6	5.4	7.6***	5.9***	6.4
Pre-existing CVD: Yes (%)	42.9	41.1	38.6	44.7*	51.9***	42.4	46.2*
Chronic diseases: Yes (%)	53.9	52.6	53.0	53.2	60.6*	54.2	53.5
Systolic BP (mean, *SD*)	134.1 (19.4)	134.2 (19.1)	133.1 (19.3)	133.8 (18.6)	136.6 (19.9)**	133.4 (19.5)	134.3 (20.8)
Diastolic BP (mean, *SD*)	80.7 (10.7)	80.7 (10.6)	81.0 (10.7)	80.6 (10.5)	81.3 (10.6)	80.6 (10.3)	80.5 (11.7)
Waist–hip ratio (mean, *SD*)	96.3 (13.7)	96.3 (13.8)*	94.8 (13.7)	96.8 (13.7)**	97.3 (13.4) **	95.8 (13.9)	96.2 (14.8)
Smoking: Present (%)	11.5	9.3	6.1	11.4	18.3***	15.8**	16.3**
Excessive alcohol: Yes (%)	10.9	11.8	14.9	10.1**	6.4***	10.1*	7.9
Physical activity: Low (%)	35.5	32.3	31.8	38.2*	41.8**	37.7*	40.9**
Depression (mean, *SD*)	4.1 (4.1)	3.8 (3.6)*	3.3 (3.2)	4.1 (3.9)*	4.8 (4.3)***	4.5 (4.0)***	4.7 (4.5)
Antihypertensives: Yes (%)	42.5	40.3	38.8	44.5*	50.5***	42.1	45.5*
Antidepressants: Yes (%)	8.9	8.1*	5.4	8.0	13.8***	11.1*	9.7
NART error score (mean, *SD*)	22.4 (11.3)	20.9 (11.1)*	14.6 (9.2)	23.7 (10.3)***	30.8 (9.8)***	24.1 (10.9)***	23.5 (11.5)*

*Notes*: The excluded sample (*N* = 1 943) is compared to the study sample (*N* = 4 620). The Stable Professional category serves as the reference level to compare social mobility groups. Ordinary least square regressions and Chi-squared tests were used as appropriate. BP = blood pressure (mmHg); CVD = cardiovascular disease; NART = National Adult Reading Test; *SD* = standard deviation.

***p < .001.

**p < .01.

*p < .05.

### The Effect of Social Mobility on Cognitive Function

The results for MoCA and MMSE from the DRMs are given in Table 2 and Supplementary Table 2, respectively. The class coefficients given in the top part of the tables indicate the class-specific deviations from the constant for the socially stable groups. The baseline model revealed a social gradient in the odds of being impaired, respectively, on the MoCA (Table 2) and MMSE (Supplementary Table 2) among the immobile groups. For instance, those who were socially stable in the professional/managerial position were significantly less likely to be cognitively impaired (odds ratio [OR] = 0.35; confidence interval [CI] = 0.26, 0.47; *p* < .001) on the MoCA whereas those stable in the semi-skilled/unskilled/never worked group were significantly more likely to be cognitively impaired (OR = 2.79; CI = 2.22, 3.49; *p* < .001). A similar social gradient in risk was evident with respect to the MMSE. The weight parameters given in the bottom part of Table 2 and Supplementary Table 2 express the relative influence of childhood and adulthood social class position on the MoCA and MMSE for those who were socially mobile. For MoCA, we see significant associations of both childhood (0.24; CI = 0.08, 0.39; *p* < .01) and adulthood SEP (0.76; CI = 0.61, 0.91; *p* < .001) with risk of cognitive impairment in later life, but adulthood has a greater relative influence. Adulthood SEP was also more influential with respect to the MMSE (0.91; CI = 0.71, 1.09; *p* < .001) whereas childhood SEP was not significant for this outcome. Subsequent models testing for mobility effects failed to find independent associations between mobility trajectories (in any direction, upward and downward) with risk of cognitive impairment, and AIC and BIC values indicated baseline models to be the most parsimonious.

**Table 2.** Logistic Diagonal Reference Models (DRMs) Estimating Social Mobility Effects on the Montreal Cognitive Assessment (MoCA)

	Baseline Model		Mobility (any direction) Model		Upward Mobility Model		Downward Mobility Model	
	OR	95% CI	OR	95% CI	OR	95% CI	OR	95% CI
Constant	0.04***	[0.03,0.05]	0.04***	[0.03,0.05]	0.04***	[0.03,0.05]	0.04***	[0.03,0.05]
Socially stable groups								
Professional/ managerial	0.35***	[0.26,0.47]	0.35***	[0.26,0.47]	0.35***	[0.26,0.47]	0.36***	[0.26,0.49]
Nonmanual/skilled manual	1.02	[0.82,1.26]	1.02	[0.82,1.26]	1.01	[0.81,1.26]	1.01	[0.81,1.27]
Semi-skilled/unskilled/never worked	2.79***	[2.22,3.49]	2.79***	[2.22,3.49]	2.80***	[2.23,3.52]	2.78***	[2.22,3.49]
Controls								
Female	1.00	[0.78,1.29]	1.00	[0.78,1.29]	1.00	[0.78,1.29]	1.00	[0.78,1.29]
Age	1.13***	[1.11,1.14]	1.13***	[1.11,1.14]	1.13***	[1.11,1.14]	1.13***	[1.11,1.14]
Childhood health	1.10	[0.97,1.25]	1.10	[0.97,1.25]	1.10	[0.97,1.24]	1.10	[0.97,1.25]
Weight parameters								
Childhood	0.24**	[0.08, 0.39]	0.24**	[0.08, 0.38]	0.21	[-0.03, 0.45]	0.22	[-0.02, 0.47]
Adulthood	0.76***	[0.61, 0.91]	0.76***	[0.61, 0.91]	0.79***	[0.54, 1.02]	0.78***	[0.53, 1.02]
Social mobility								
Mobile			1.01	[0.79,1.30]				
Upward					1.07	[0.67,1.70]		
Downward							0.96	[0.59,1.58]
AIC	1 888		1 890		1 890		1 890	
BIC	1 933		1 942		1 942		1 942	
Degrees of freedom	7		8		8		8	
Observations	4 620		4 620		4 620		4 620	

*Notes*: The Constant denotes average odds for a male of average age and average childhood health. Socially stable groups: ORs are given for each socially stable group. They indicate the class-specific deviations from the constant. Controls: All models are adjusted for age, sex, and childhood health. Weight parameters: Bounded between 0 and 1, they express the relative contribution of childhood SEP and adulthood SEP on MoCA for the socially mobile. A high value of the childhood weight means that childhood class has greater influence on MoCA, whereas a high weight for adulthood means that adulthood class has greater influence on MoCA. The mobility (any direction), upward mobility and downward mobility, models test the *independent* effect of social mobility on MoCA after accounting for the relative importance of childhood and adulthood SEP. 95% CIs are given in brackets. AIC = Akaike Information Criteria; BIC = Bayesian Information Criteria; CI = confidence interval; OR = odds ratio; SEP = socioeconomic position.

***p < .001.

**p < .01.

*p < .05.

### The Effect of Social Mobility on Brain Structure

In comparison with cognitive function, the DRMs for brain structure were not as clearly socially patterned although those stable in the semi-skilled/unskilled/never worked groups had smaller total GMV (*b* = −6 759.3; CI = −12 103.2, −1 415.4; *p* = .01; Table 3) and smaller AC volume (*b* = −362.8; CI = −575.6, −150.1; *p* = .001; Table 4). In addition, those who were socially stable in the professional/managerial position had larger vmPFC volume (*b* = 158.3; CI = 28.1, 288.4; *p* = .01; Table 5). Among the socially mobile, adulthood SEP had a greater influence on total GMV (0.77; CI = 0.28, 1.25; *p* = .002) and the AC volume (0.76; CI = 0.38, 1.12; *p* < .001) whereas childhood SEP was more influential for the vmPFC (1.00; CI = 0.34, 1.68; *p* = .003). Our models failed to find an independent association of mobility trajectories (in any direction, upward and downward) with the brain structure outcomes (Tables 3–5). No association of childhood or adulthood SEP with cortical thickness, hippocampal, or amygdala volumes was observed (results not shown).

**Table 3.** Point Estimates From Diagonal Reference Models (DRMs) Estimating Social Mobility Effects on Total Grey Matter Volume (GMV)

	Baseline Model		Mobility (any direction) Model		Upward Mobility Model		Downward Mobility Model	
	*b*	95% CI	*b*	95% CI	*b*	95% CI	*b*	95% CI
Constant	593 131***	[588 796, 597 467]	592 793***	[587 657, 597 929]	593 222***	[588 404, 598 040]	592 723***	[588 059, 597 386]
Socially stable groups								
Professional/managerial	3 178.2	[−1 325.5, 7 682.0]	3 229.3	[−1 347.4, 7 806.2]	3 178.9	[−1 267.8, 7 625.7]	3 348.8	[−1 024.9, 7 722.5]
Nonmanual/skilled manual	3 581.0	[−827.6, 7 989.8]	3 578.2	[−893.5, 8 049.9]	3 586.3	[−763.0, 7 935.6]	3 597.5	[−612.4, 7 807.4]
Semi-skilled/unskilled/never worked	−6 759.3**	[−12 103.2, −1 415.4]	−6 807.5**	[−12 161.8, −1 453.3]	−6 765.2**	[−12 111.7, −1 418.7]	−6 946.3**	[−12 339.7, −1 552.9]
Controls								
Female	−18 676.1***	[−25 000.1, −12 352.1]	−18 725.8***	[−25 062.3, −12 389.2]	−18 676.1***	[−25 000.1, −12 352.1]	−18 786.3***	[−25 125.5, −12 447.2]
Age	−2 792.2***	[−3 146.5, −2 437.9]	−2 793.5***	[−3 147.9, −2 439.1]	—2 792.2***	[-3 146.5, -2 437.9]	—2 783.7***	[−3 139.7, −2 427.6]
eTIV	0.1***	[0.01, 0.02]	0.2***	[0.01, 0.02]	0.1***	[0.01, 0.02]	0.1***	[0.01, 0.02]
Childhood health	−999.0	[−37 96.4, 1 798.2]	−1 008.9	[−3 807.1, 1 789.3]	−999.0	[−3 796.4, 1 798.2]	−1 026.8	[−3 825.9, 1 772.1]
Weight parameters								
Childhood	0.23	[−0.25, 0.71]	0.24	[−0.25, 0.71]	0.21	[−0.38, 0.81]	0.17	[−0.36, 0.70]
Adulthood	0.77**	[0.28, 1.25]	0.76**	[0.28, 1.25]	0.79*	[0.18, 1.38]	0.83**	[0.29, 1.36]
Mobility status								
Mobile			650.1	[−4 645.2, 5 945.6]				
Upward					−290.4	[−7 016.6, 6 435.7]		
Downward							1 952.7	[−6 230.1, 10 135.5]
AIC	10 828		10 830		10 830		10 830	
BIC	10 865		10 871		10 871		10 871	
Degrees of freedom	9		10		10		10	
Observations	464		464		464		464	

*Notes*: The Constant denotes average total GMV for a male of average age, average childhood health and average eTIV. Socially stable groups: the class coefficients indicate the class-specific deviations from the constant. Controls: all models are adjusted for age, sex, eTIV and childhood health. Weight parameters: bounded between 0 and 1, they express the relative contribution of childhood SEP and adulthood SEP on total GMV for the socially mobile. A high value of the childhood weight means that childhood class has greater influence on GMV, whereas a high weight for adulthood means that adulthood class has greater influence on GMV. The mobility (any direction), upward mobility and downward mobility models test the *independent* effect of social mobility on total GMV after accounting for the relative importance of childhood and adulthood SEP. 95% CIs are given in brackets. AIC = Akaike Information Criteria; BIC = Bayesian Information Criteria; CI = confidence interval; eTIV = estimated intracranial volume; OR = odds ratio; SEP = socioeconomic position.

***p < .001.

**p < .01.

*p < .05.

**Table 4.** Point Estimates From Diagonal Reference Models (DRMs) Estimating Social Mobility Effects on the Anterior Cingulate (AC) Volume

	Baseline Model		Mobility (any direction) Model		Upward Mobility Model		Downward Mobility Model	
	*b*	95% CI	*b*	95% CI	*b*	95% CI	*b*	95% CI
Constant	8 077***	[7 905, 8 249]	8 089***	[7 885, 8 292]	8 039***	[7 848, 8 230]	8 125***	[7 943, 8 307]
Socially stable groups								
Professional/managerial	176.4*	[6.6, 346.3]	177.5*	[8.6, 346.2]	145.6	[−44.1, 335.4]	125.8	[−67.0, 318.7]
Nonmanual/skilled manual	186.4	[−8.9, 381.7]	183.4	[−14.4, 381.2]	215.7*	[20.9, 410.5]	216.9*	[25.4, 408.3]
Semi-skilled/unskilled/never worked	−362.8***	[−575.6, −150.1]	−360.8***	[−574.9, −146.9]	−361.4***	[−572.7, −150.1]	−342.7***	[−556.5, −129.0]
Controls								
Female	−31.9	[−282.3, 218.4]	−30.2	[−281.1, 220.6]	−33.7	[−284.1, 216.5]	−17.9	[−268.6, 232.6]
Age	−30.7***	[−44.7, −16.7]	−30.7***	[−44.7, −16.7]	−31.3***	[−45.3, −17.2]	−31.5***	[−45.6, −20.4]
eTIV	0.00***	[0.00, 0.00]	0.00***	[0.00, 0.00]	0.00***	[0.00, 0.00]	0.00***	[0.00, 0.00]
Childhood health	−34.9	[−145.7, 75.8]	−34.9	[−145.7, 76.3]	−36.1	[−146.8, 74.5]	−32.78	[−143.4, 77.8]
Weight parameters								
Childhood	0.24	[−0.12, 0.61]	0.23	[−0.16, 0.62]	0.39	[−0.04, 0.81]	0.41*	[0.01, 0.82]
Adulthood	0.76***	[0.38, 1.12]	0.77***	[0.38, 1.16]	0.61***	[0.18, 1.04]	0.59***	[0.18, 0.99]
Mobility status								
Mobile			−22.8	[−233.9, 188.2]				
Upward					123.9	[−139.9, 387.7]		
Downward							−228.4	[−542.3, 85.6]
AIC	7 832		7 833		7 833		7 832	
BIC	7 869		7 875		7 875		7 973	
Degrees of freedom	9		10		10		10	
Observations	464		464		464		464	

*Notes*: The Constant denotes average AC volume for a male of average age, average childhood health and average eTIV. Socially stable groups: the class coefficients indicate the class-specific deviations from the constant. Controls: all models are adjusted for age, sex, eTIV, and childhood health. Weight parameters: bounded between 0 and 1, they express the relative contribution of childhood SEP and adulthood SEP on the AC volume for the socially mobile. A high value of the childhood weight means that childhood class has greater influence on the AC, whereas a high weight for adulthood means that adulthood class has greater influence on the AC. The mobility (any direction), upward mobility and downward mobility, models test the *independent* effect of social mobility on the AC volume after accounting for the relative importance of childhood and adulthood SEP. 95% CIs are given in brackets. AIC: Akaike Information Criteria; BIC: Bayesian Information Criteria; CI = confidence interval; eTIV = estimated intracranial volume; OR = odds ratio; SEP = socioeconomic position.

***p < .001 . *p < .05.

**Table 5.** Point Estimates From Diagonal Reference Models (DRMs) Estimating Social Mobility Effects on the Ventromedial Prefrontal Cortex (vmPFC) Volume

	Baseline Model		Mobility (any direction) Model		Upward Mobility Model		Downward Mobility Model	
	*b*	95% CI	*b*	95% CI	*b*	95% CI	*b*	95% CI
Constant	10 496***	[10 377, 10 615]	10 494***	[10 353, 10 635]	10 453***	[10 317, 10 589]	10 531***	[10 401, 10 661]
Socially stable groups								
Professional/ managerial	158.3**	[28.1, 288.4]	158.7**	[27.5, 289.9]	176.9**	[46.5, 307.3]	160.5**	[32.5, 288.6]
Nonmanual/skilled manual	−68.4	[−162.7, 25.8]	−68.4	[−162.6, 25.6]	−65.1	[−143.7, 13.6]	−64.8	[−145.6, 15.8]
Semi-skilled/unskilled/never worked	−89.8	[−212.9, 33.3]	−90.3	[−214.3, 33.7]	−111.8*	[−220.6, −3.05]	−95.6	[−199.0, 7.72]
Controls								
Female	−620.8***	[−794.9, −446.8]	−621.2***	[−795.7, −446.6]	−627.4***	[−801.5, −453.4]	−614.4***	[−788.5, −440.4]
Age	−41.1***	[−50.9, −31.3]	−41.1***	[−50.9, −31.3]	−41.3***	[−51.0, −31.5]	−41.3***	[−51.0, −31.5]
eTIV	0.0***	[0.00, 0.00]	0.0***	[0.00, 0.00]	0.0***	[0.00, 0.00]	0.0***	[0.00, 0.00]
Childhood health	−9.7	[−86.7, 67.3]	−9.7	[−86.7, 67.3]	−9.8	[−86.7, 66.9]	−7.2	[−84.1, 69.7]
Weight parameters								
Childhood	1.00**	[0.34, 1.68]	1.01**	[0.34, 1.68]	1.30**	[0.46, 2.14]	1.27**	[0.40, 2.14]
Adulthood	−0.00	[−0.68, 0.66]	−0.01	[−0.68, 0.66]	−0.30	[−1.14, 0.53]	−0.27	[−1.14, 0.59]
Mobility status								
Mobile			3.9	[−143.4, 151.2]				
Upward					148.5	[−85.7, 382.8]		
Downward							−161.7	[−406.3, 82.9]
AIC	7 495		7 496		7 494		7 495	
BIC	7 532		7 537		7 536		7 536	
Degrees of freedom	9		10		10		24	
Observations	464		464		464		464	

*Notes*: The Constant denotes average vmPFC volume for a male of average age, average childhood health and average eTIV. Socially stable groups: the class coefficients indicate the class-specific deviations from the constant. Controls: all models are adjusted for age, sex, eTIV, and childhood health. Weight parameters: bounded between 0 and 1, they express the relative contribution of childhood SEP and adulthood SEP on the vmPFC volume for the socially mobile. A high value of the childhood weight means that childhood class has greater influence on the vmPFC, whereas a high weight for adulthood means that adulthood class has greater influence on the vmPFC. The mobility (any direction), upward mobility and downward mobility, models test the *independent* effect of social mobility on the vmPFC volume after accounting for the relative importance of childhood and adulthood SEP. 95% CIs are given in brackets. AIC = Akaike Information Criteria; BIC = Bayesian Information Criteria; CI = confidence interval; eTIV = estimated intracranial volume; OR = odds ratio; SEP = socioeconomic position.

***p < .001. **p < .01. *p < .05.

### Sensitivity Analyses Using DRMs and Further Controlling for Diseases, Lifestyle, Medications, and Premorbid Intelligence

Sensitivity analyses showed the same social gradient in late-life cognitive function (MoCA, Supplementary Table 3], and MMSE, Supplementary Table 4) and brain structure (vmPFC, Supplementary 5; AC, Supplementary Table 6) when the DRMs were further adjusted for diseases and lifestyle factors. The association between adulthood SEP and total GMV was borderline significant (*p* = .06; Supplementary Table 7). When further adjusted for NART, the DRMs revealed no effect of childhood or adulthood SEP on cognitive function (for both MoCA and MMSE, Supplementary Tables 8 and 9) nor with total GMV (Supplementary Table 10). There were no differences in vmPFC volume between the socially stable classes; however, childhood SEP was significantly more influential on the vmPFC volume (1.31, 95% CI = 0.01, 2.60, *p* = .04) for the socially mobile (Supplementary Table 11). Adding NART as an additional covariate did not substantially change the strength of the SEP–AC volume relationship (Supplementary Table 12). Higher adulthood SEP was associated with larger AC volume (*p* < .05) and was the most influential for AC volume (0.73, 95% CI = 0.44, 1.01, *p* < .001). Interaction terms by sex were not significant.

### Sensitivity Analyses Using the Traditional Approach

Regression models examining the effect of social mobility on cognitive function (Supplementary Table 13) showed that all groups had higher probability of being impaired on the MoCA (Supplementary Figure 2, Panel A) and MMSE (Supplementary Figure 2, Panel B) compared to the stable professional/managerial reference group. The upwardly mobile ranked intermediate between the stable professional/managerial group and the stable nonmanual/skilled manual group, and the downwardly mobile ranked intermediate between the stable nonmanual/skilled manual group and the stable semi-skilled/unskilled/never worked group.

Regression models examining the effect of social mobility on brain structure (Supplementary Table 14) indicated smaller total GMV for the stable semi-skilled/unskilled/never worked group compared to the stable professional/managerial (Supplementary Figure 3, Panel A). The stable semi-skilled/unskilled/never worked group and the upwardly mobile also had lower vmPFC volumes compared to the stable professional/managerial group, with the upwardly mobile ranking between the stable semi-skilled/unskilled/never worked and the stable nonmanual/skilled manual group and the downwardly mobile ranking between the stable nonmanual/skilled manual group and the stable professional/managerial group (Supplementary Figure 3, Panel B). AC volume was lower for the downwardly mobile and the stable semi-skilled/unskilled/never worked group compared to the stable professional/managerial (Supplementary Figure 3, Panel C). Social mobility was not associated with cortical thickness, nor with hippocampal or amygdala volumes.

## Discussion

This study explored whether the experience of intergenerational social mobility was associated with deficits in cognitive status and brain structural indices among a large representative sample of community-dwelling older adults, using an innovative methodological approach that allows us to isolate the mobility effect distinct from childhood and adulthood SEP. We observed a social gradient in global cognitive functioning among those who were intergenerationally stable in their social class position implying that life-course SEP may help determine risk for dementia in later life. Although this is consistent with what previous studies have shown (39), DRM allows us to put parameter estimates on the relative contribution of childhood and adulthood SEP to these later-life differences in cognitive functioning. The analysis revealed that both childhood and adulthood SEP were significantly associated with cognitive status on the MoCA among the socially mobile, but adulthood predominates in terms of its influence. Results were similar for the MMSE with contributions for adulthood SEP, but not for childhood SEP in this instance.

Our analysis revealed no independent association of mobility in any direction with cognitive status as assessed using the MoCA and MMSE, a finding that implies that the linear combination of childhood and adulthood SEP is sufficient for capturing the life-course effects of SEP on later-life cognitive status. Hence our results are compatible with the accumulation model which suggests that SEP is compounding in terms of its influence on cognition. Consistent with this interpretation, the cross-classification of childhood and adulthood SEP in our sensitivity analyses indicated that the upwardly and downwardly mobile groups ranked intermediate between their childhood class and their adulthood class, and that the upwardly mobile has better outcomes than the downwardly mobile with the exception of the vmPFC. This implies that mobility dilutes the effect of childhood SEP, and the outcome is more strongly determined by the class group they join in later life, which is why the upwardly mobile perform better than the downwardly mobile, but not as well as those who were stable high in the professional/managerial position across the life course. Table 1 provides additional insight as to why these patterns emerge as the health and lifestyle profile of the upwardly mobile is more akin to those who were intergenerationally stable in the professional/managerial class, whereas the downwardly mobile resemble those who were stable at lower occupational positions. This process of assortment has been labeled “gradient constraint” (40), and the results are compatible with what has been observed for other physical and mental health outcomes (41).

We observed similar patterns in a brain region implicated in cognitive aging, the vmPFC, and in a region previously reported to be sensitive to SEP, the AC, and total GMV. However, there were some nuances in terms of the relative weights to be afforded to childhood and adulthood SEP. Higher weights were observed for adulthood SEP with respect to GMV and AC volume, but not for the vmPFC, where childhood SEP was much more influential. The prefrontal cortex undergoes a prolonged maturation period in early development which plausibly increases its susceptibility to environmental changes (42). Further, in comparison to other cortical regions, the prefrontal cortex experiences large volumetric changes both in development and in aging (43). Accordingly, although our lack of longitudinal data disqualifies us from asserting the timeline of SEP–brain differences, our findings do cautiously raise the hypothesis that this region may be particularly sensitive to socioeconomic influences in early life. Of note, although others have observed increased cortical thinning and reduced hippocampal volume in individuals from lower SEP (12,15,44), the current study found no evidence of an association with cortical thickness or the hippocampus. In children, lower SEP has been linked to smaller amygdala volume, spurring hypotheses regarding stress exposure as a potential mechanistic explanation for this relationship. In contrast, we did not observe SEP differences in the amygdala in late adulthood.

Finally, controlling for premorbid intelligence using the NART nullified the association between SEP and cognitive status, but it did not alter the relationship between SEP and regional measures of brain structure. Given the well-established interrelation between cognitive screening tools and the NART, this is perhaps unsurprising (45). Of course, one could argue that childhood general cognitive ability (GCA) could be responsible for both selection into higher SEP categories and offset risk for cognitive decline in later life. For example, in the Aberdeen birth cohort (46), childhood GCA predicted adulthood SEP more strongly than childhood SEP, and other studies from the same group have shown that GCA measured at age 11 reduces the risk for cause-specific (including dementia) and all-cause mortality (47). Unfortunately, TILDA does not have a measure of childhood GCA, so we cannot rule out these competing causal explanations for the observed social gradients. Still, these findings highlight the added benefit of using volumetric brain measures, which are less confounded by premorbid intelligence estimates, to identify differences related to SEP.

We acknowledge a number of limitations. First, although the TILDA study recruited a sample that was representative of the Irish adult population, the MRI sample did present a higher prevalence of “Stable Professionals” than the parent cohort (MRI sample: 16% vs TILDA cohort: 11.5%). Second, the SEP–brain–cognition associations reported here are unlikely to be representative of countries with different SEP distributions, as other studies (13) showed that SEP was more strongly related to gray matter and cognitive measures in the U.S. than in European cohorts. Third, with a reduced sample size in the MRI sample, relative to the TILDA cohort, it is possible that the MRI analysis was underpowered to detect SEP-related differences. However, although modest, our sample size is in keeping with the SEP-MRI literature, and comparable sample sizes have revealed SEP-level differences in brain structures (14). Fourth, our cognitive outcomes were derived from two cognitive screening instruments: the MoCA and the MMSE. These tools can provide a snapshot of cognitive status, but they do not provide a comprehensive neuropsychological evaluation sufficient to diagnose a dementia disorder. It would, therefore, be desirable, to replicate our findings in data sets with more extensive cognitive profiling. Fifth, although we have interpreted the results as being consistent with an accumulation model, one could argue that they are also consistent with a recency effect as adulthood SEP had a higher relative influence on the outcomes for the socially mobile across almost all outcomes. Relatedly, it should be acknowledged that our definition of intergenerational mobility is constrained to only 2 time points which means we may be missing substantial intragenerational transitions in a person’s mobility trajectory over time. Future longitudinal studies with repeated measures of SEP at frequent points across the life span are required to further characterize mobility trajectories and choose between competing life-course models using structured life-course modeling approaches (48). In addition, we could not take account of the degree of mobility (ie, the number of positions someone is mobile relative to their childhood class) in the analysis due to relatively small numbers in the MRI group and the fact that we aggregated social class groups into 3 categories. Finally, it should be acknowledged that current DRMs are not optimized for undertaking mediation analysis to elucidate the pathways through which mobility affects the outcome of interest.

## Conclusion

This study explored the role of life-course SEP and social mobility on 2 clinically relevant indices of cognitive status and MRI-derived markers of brain structure using DRMs. This is the first study to employ DRM methodology to this end. Our results indicate that lower SEP is associated with an increased risk for cognitive impairment, as well as reduced volumetric measures of total GMV, AC, and vmPFC in late adulthood. Social mobility does not have a direct effect on brain function or structure, per se, but seems to operate indirectly with the mobile adopting the characteristics of the class they join while carrying some of the legacy effect of childhood. Taken in conjunction, our results suggest that both childhood and adulthood SEP are important in shaping later-life cognition, but that adulthood SEP predominates in terms of its influence. Nevertheless, it should be acknowledged that childhood SEP sets constraints on adulthood SEP and is part of longer-term chains of risk that affect brain structure and function through multiple dynamic and interacting etiologic pathways, including material deprivation, psychosocial stress, lifestyle behaviors, and environmental exposures. From a social policy standpoint, this is potentially an important insight as it suggests that the outcome is modifiable if socioeconomic circumstances change. When, where, and how one intervenes to offset riskier long-term outcomes associated with low SEP across the life course remains a matter of conjecture.

## Supplementary Material

glad068_suppl_Supplementary_Material
