# Supplementary material for: Examining the Impact of Socioeconomic Position Across the Life Course on Cognitive Function and Brain Structure in Healthy Aging
Source: J Gerontol A Biol Sci Med Sci. 2023 Feb 23;78(6):890–901. doi: 10.1093/gerona/glad068 (PMC10235205; doi:10.1093/gerona/glad068)
Supplement: glad068_suppl_Supplementary_Material [file glad068_suppl_supplementary_material.docx]

**SUPPLEMENT MATERIAL**

**Examining the impact of socioeconomic position (SEP) across the life course on cognitive function and brain structure in healthy ageing**

**Figure S1.** Flow diagram of the main study sample and the MRI sub-sample.

**
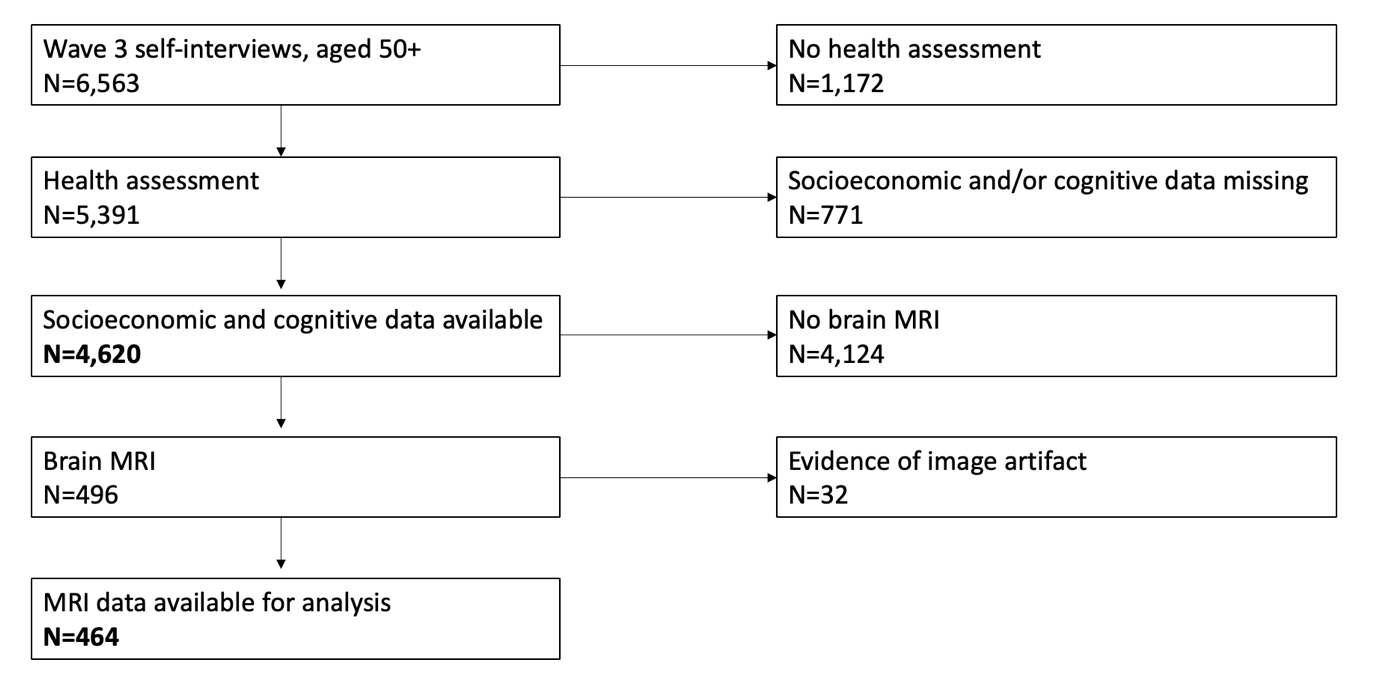
**

**Figure S2.** Conditional probability of impairment on the Montreal Cognitive Assessment (MoCA) (Panel A) and the Mini-Mental State Examination (MMSE) (Panel B) according to social mobility status, using binary logistic regression.

Panel A Panel B

Note. Adjusted for age, sex and childhood health. Stable prof / man = stable professional/managerial group; Stable non-man / skilled man = stable non-manual/skilled manual group; Stable semi-skilled / unskilled = stable semi-skilled/unskilled/never worked group.

**Figure S3.** Conditional means of total grey matter volume (GMV) (Panel A), ventro-medial prefrontal cortex (vmPFC) volume (Panel B) and anterior cingulate (AC) volume (Panel C) according to social mobility status, using ordinary least squares regression.

Panel A Panel B Panel C

Note. Adjusted for age, sex childhood health and estimated Intracranial Volume (eTIV). Stable prof / man = stable professional/managerial group; Stable non-man / skilled man = stable non-manual/skilled manual group; Stable semi-skilled / unskilled = stable semi-skilled/unskilled/never worked group.

**Table S1.** Descriptive characteristics of the MRI sample (N=464) overall, per social mobility category and as compared to the excluded participants (N=6,099).

|  | **Study sample**  **(N=464)** | **Upwardly mobile (N=190)** | **Stable Professional (N=76)** | **Stable**  **Non-manual/ Skilled manual (N=78)** | **Stable Unskilled/ Never worked (N=40)** | **Downwardly mobile (N=80)** | **Excluded**  **(N=6,099)** |
| --- | --- | --- | --- | --- | --- | --- | --- |
| **Age** (mean, SD) | 68.44 (7.4) | 69.5 (6.9) | 68.9 (7.7) | 67.8 (7.3) | 66.9 (6.7) | 66.6 (8.2) | 66.2 (9.5)^***^ |
| **Gender:** Female (%) | 53.5 | 51.6 | 47.4 | 48.7 | 62.5 | 63.7^*^ | 55.9 |
| **Childhood health:** Fair/poor (%) | 5.8 | 4.2 | 9.2 | 6.4 | 5.0 | 6.3 | 6.0 |
| **Pre-existing CVD:** Yes (%) | 44.2 | 40.5 | 52.6 | 39.7 | 57.5 | 42.5 | 43.9^*^ |
| **Chronic diseases:** Yes (%) | 56.5 | 60.5 | 57.9 | 44.9 | 62.5 | 53.7 | 53.5 |
| **Systolic BP** (mean, SD) | 134.2 (18.7) | 135.2 (18.8) | 133.7 (20.6) | 132.8 (15.2) | 138.6 (21.1) | 132.1 (17.5) | 134.1 (19.5) |
| **Diastolic BP** (mean, SD) | 80.1 (10.3) | 80.7 (10.6) | 81.0 (10.7) | 80.6 (10.5) | 81.3 (10.6) | 80.6 (10.3) | 80.8 (10.7) |
| **Waist hip ratio** (mean, SD) | 95.6 (13.1) | 96.4 (12.7) | 94.1 (12.3) | 97.7 (14.9) | 92.5 (13.3) | 94.5 (12.5) | 96.3 (13.9) |
| **Smoking:** Present (%) | 6.0 | 5.3 | 0.0 | 3.8 | 17.5 | 10.0 | 13.4^**^ |
| **Excessive alcohol:** yes (%) | 10.9 | 8.9 | 12.8 | 10.0 | 6.4 | 3.7 | 10.1 |
| **Physical activity:** Low (%) | 34.0 | 32.1 | 25.0 | 37.2 | 45.0 | 38.7 | 37.3^**^ |
| **Depression** (mean, SD) | 3.6 (3.4) | 3.0 (2.9) | 3.6 (3.5) | 3.7 (3.5) | 4.6 (4.4) | 4.1 (3.5) | 4.2 (3.9) |
| **Antihypertensives:** Yes (%) | 42.0 | 40.0 | 51.3 | 43.6 | 45.0 | 35.0^*^ | 43.5^*^ |
| **Antidepressants:** Yes (%) | 6.7 | 5.3 | 3.9 | 8.9 | 7.5 | 10.0 | 9.4 |
| **NART error score** (mean, SD) | 20.4 (11.2) | 19.2 (10.8)^***^ | 13.2 (7.8) | 22.6 (10.3)^***^ | 31.0 (9.3)^***^ | 22.7 (11.7)^***^ | 22.8 (11.3)^*^ |

Note. BP = Blood pressure (mmHg); NART = National Adult Reading Test. *** p<.001; ** p<.01; * p<.05. The excluded sample (N=6,099) is compared to the study sample (N=464). The Stable Professional category serves as the reference level to compare social mobility groups. Ordinary-least square regressions and Chi-squared tests were used as appropriate.

**Table S2.** Logistic diagonal reference models (DRMs) estimating social mobility effects on the Mini Mental State Examination (MMSE).

|  | ***Baseline model*** |  | ***Mobility***  ***(any direction) model*** |  | ***Upward mobility model*** | | ***Downward mobility model*** | | | |
| --- | --- | --- | --- | --- | --- | --- | --- | --- | --- | --- |
|  | OR | *95% CI* | OR | *95% CI* | OR | *95% CI* | | OR | | *95% CI* |
| Constant | 0.04^***^ | [0.03,0.05] | 0.03^***^ | [0.02,0.05] | 0.03^***^ | [0.03,0.05] | | | 0.03^***^ | [0.02,0.05] |
| **Socially stable groups** |  |  |  |  |  |  | | |  |  |
| Professional / managerial | 0.35^***^ | [0.25, 0.50] | 0.34^***^ | [0.24, 0.49] | 0.35^***^ | [0.25, 0.50] | | | 0.32^***^ | [0.21, 0.49] |
| Non-manual /skilled manual | 1.06 | [0.84, 1.33] | 1.07 | [0.85, 1.34] | 1.03 | [0.82, 1.29] | | | 1.13 | [0.83, 1.55] |
| Semi-skilled/unskilled/never worked | 2.70^***^ | [2.10, 3.47] | 2.75^***^ | [2.10, 3.47] | 2.74^***^ | [2.10, 3.47] | | | 2.75^***^ | [2.10, 3.47] |
| **Controls** |  |  |  |  |  |  | | |  |  |
| Female | 0.84 | [0.64, 1.11] | 0.84 | [0.64, 1.12] | 0.84 | [0.64, 1.11] | | | 0.85 | [0.64, 1.12] |
| Age | 1.10^***^ | [1.08, 1.11] | 1.10^***^ | [1.08, 1.11] | 1.10^***^ | [1.08, 1.11] | | | 1.10^***^ | [1.08, 1.11] |
| Childhood health | 1.34^***^ | [1.18, 1.52] | 1.33^***^ | [1.17, 1.52] | 1.34^***^ | [1.17, 1.52] | | | 1.33^***^ | [1.17, 1.52] |
| **Weight parameters** |  |  |  |  |  |  | | |  |  |
| Childhood | 0.09 | [-0.09, 0.28] | 0.10 | [-0.08, 0.28] | 0.02 | [-0.26, 0.30] | | | 0.21 | [-0.26, 0.30] |
| Adulthood | 0.91^***^ | [0.71, 1.09] | 0.90^***^ | [0.71, 1.08] | 0.98^***^ | [0.69, 1.26] | | | 0.79^***^ | [0.46, 1.11] |
| **Mobility status** |  |  |  |  |  |  | | |  |  |
| Mobile |  |  | 1.12 | [0.84, 1.48] |  |  | | |  |  |
| Upward |  |  |  |  | 1.21 | [0.71, 2.06] | | |  |  |
| Downward |  |  |  |  |  |  | | | 1.29 | [0.70, 2.37] |
| AIC | 1589 |  | 1591 |  | 1591 |  | | | 1591 |  |
| BIC | 1634 |  | 1642 |  | 1642 |  | | | 1642 |  |
| Degrees of freedom | 7 |  | 8 |  | 8 |  | | | 8 |  |
| Observations | 4620 |  | 4620 |  | 4620 |  | | | 4620 |  |

Note. *OR* = Odds Ratios. The *Constant* denotes average odds for a male of average age and average childhood health. *Socially stable groups*: OR are given for each socially stable group. They indicate the class specific deviations from the constant. *Controls*: all models are adjusted for age, sex, and childhood health. *Weight parameters*: bounded between 0 and 1, they express the relative contribution of childhood SEP and adulthood SEP on MMSE for the socially mobile. A high value of the childhood weight means that childhood class has greater influence on MMSE, while a high weight for adulthood means that adulthood class has greater influence on MMSE. The mobility (any direction), upward mobility and downward mobility models test the *independent* effect of social mobility on MMSE after accounting for the relative importance of childhood and adulthood SEP. 95% confidence intervals (CI) are given in brackets. AIC: Akaike Information Criteria. BIC: Bayesian Information Criteria. *** p<.001; ** p<.01; * p<.05.

**Table S3.** Logistic diagonal reference models (DRMs) estimating social mobility effects on the Montreal Cognitive Assessment (MoCA) with additional adjustment for lifestyle and disease factors.

|  | ***Baseline model*** |  | ***Mobility***  ***(any direction) model*** |  | ***Upward mobility model*** | | ***Downward mobility model*** | | | |
| --- | --- | --- | --- | --- | --- | --- | --- | --- | --- | --- |
|  | OR | *95% CI* | OR | *95% CI* | OR | *95% CI* | | OR | | *95% CI* |
| Constant | 0.17^***^ | [0.04, 0.72] | 0.17^***^ | [0.04, 0.72] | 0.16^***^ | [0.04, 0.71] | | | 0.17^***^ | [0.04, 0.73] |
| **Socially stable groups** |  |  |  |  |  |  | | |  |  |
| Professional / managerial | 0.38^***^ | [0.28, 0.51] | 0.37^***^ | [0.28, 0.51] | 0.38^***^ | [0.28, 0.51] | | | 0.38^***^ | [0.28, 0.52] |
| Non-manual /skilled manual | 1.03 | [0.83, 1.29] | 1.04 | [0.83, 1.29] | 1.02 | [0.82, 1.28] | | | 1.02 | [0.81, 1.29] |
| Semi-skilled/unskilled/never worked | 2.58^***^ | [2.03, 3.26] | 2.58^***^ | [2.03, 3.28] | 2.60^***^ | [2.04, 3.30] | | | 2.57^***^ | [2.03, 3.25] |
| **Controls** |  |  |  |  |  |  | | |  |  |
| Female | 0.75 | [0.56, 1.03] | 0.75 | [0.56, 1.02] | 0.75 | [0.55, 1.02] | | | 0.75 | [0.56, 1.03] |
| Age | 1.13^***^ | [1.11, 1.14] | 1.13^***^ | [1.11, 1.14] | 1.13^***^ | [1.11, 1.14] | | | 1.13^***^ | [1.11, 1.14] |
| Childhood health | 1.03 | [0.90, 1.17] | 1.03 | [0.90, 1.17] | 1.03 | [0.90, 1.17] | | | 1.03 | [0.90, 1.17] |
| Current smoker | 1.51^*^ | [1.01, 2.26] | 1.51^*^ | [1.01, 2.26] | 1.51^*^ | [1.01, 2.25] | | | 1.51^*^ | [1.01, 2.26] |
| Excessive alcohol | 0.97 | [0.57, 1.66] | 0.97 | [0.57, 1.66] | 0.97 | [0.57, 1.66] | | | 0.97 | [0.57, 1.66] |
| Waist-Hip ratio | 0.99 | [0.98, 1.01] | 0.99 | [0.98, 1.01] | 0.99 | [0.98, 1.01] | | | 0.99 | [0.98, 1.01] |
| Chronic diseases | 0.94 | [0.71, 1.25] | 0.94 | [0.71, 1.25] | 0.94 | [0.71, 1.25] | | | 0.94 | [0.71, 1.25] |
| Cardiovascular diseases | 1.20 | [0.86, 1.68] | 1.20 | [0.86, 1.68] | 1.20 | [0.86, 1.68] | | | 1.20 | [0.86, 1.67] |
| Systolic blood pressure | 0.99^*^ | [0.98, 1.00] | 0.99^*^ | [0.98, 1.00] | 0.99^*^ | [0.98, 1.00] | | | 0.99^*^ | [0.98, 1.00] |
| Diastolic blood pressure | 1.00 | [0.99, 1.02] | 1.00 | [0.99, 1.02] | 1.00 | [0.99, 1.02] | | | 1.00 | [0.99, 1.02] |
| High levels of physical activity | 0.60^**^ | [0.41, 0.87] | 0.60^**^ | [0.41, 0.87] | 0.60^**^ | [0.41, 0.87] | | | 0.60^**^ | [0.41, 0.87] |
| Depression | 1.06^***^ | [1.03, 1.09] | 1.06^***^ | [1.03, 1.09] | 1.06^***^ | [1.03, 1.09] | | | 1.06^***^ | [1.03, 1.09] |
| Antidepressants | 1.89^***^ | [1.34, 2.67] | 1.89^***^ | [1.34, 2.67] | 1.90^***^ | [1.34, 2.68] | | | 1.90^***^ | [1.34, 2.68] |
| Antihypertensives | 0.77 | [0.55, 1.08] | 0.77 | [0.55, 1.08] | 0.77 | [0.55, 1.09] | | | 0.77 | [0.55, 1.08] |
| **Weight parameters** |  |  |  |  |  |  | | |  |  |
| Childhood | 0.25^**^ | [0.08, 0.41] | 0.25^**^ | [0.08, 0.41] | 0.21 | [-0.05, 0.47] | | | 0.23^**^ | [0.08, 0.41] |
| Adulthood | 0.75^***^ | [0.58, 0.91] | 0.75^***^ | [0.59, 0.92] | 0.79^***^ | [0.53, 1.05] | | | 0.77^***^ | [0.58, 0.91] |
| **Mobility status** |  |  |  |  |  |  | | |  |  |
| Mobile |  |  | 1.01 | [0.78, 1.30] |  |  | | |  |  |
| Upward |  |  |  |  | 1.09 | [0.67,1.76] | | |  |  |
| Downward |  |  |  |  |  |  | | | 0.94 | [0.56,1.55] |
| AIC | 1811 |  | 1813 |  | 1813 |  | | | 1813 |  |
| BIC | 1952 |  | 1960 |  | 1961 |  | | | 1961 |  |
| Degrees of freedom | 22 |  | 23 |  | 23 |  | | | 23 |  |
| Observations | 4620 |  | 4620 |  | 4620 |  | | | 4620 |  |

Note. *OR* = Odds Ratios. The *Constant* denotes average odds for a male of average age and average childhood health. *Socially stable groups*: OR are given for each socially stable group. They indicate the class specific deviations from the constant. *Controls*: all models are adjusted for age, sex, childhood health, cardiovascular diseases, chronic diseases, blood pressure, waist hip ratio, smoking status, alcohol consumption, physical activity, depression, antidepressants and antihypertensive medications. *Weight parameters*: bounded between 0 and 1, they express the relative contribution of childhood SEP and adulthood SEP on MoCA for the socially mobile. A high value of the childhood weight means that childhood class has greater influence on MoCA, while a high weight for adulthood means that adulthood class has greater influence on MoCA. The mobility (any direction), upward mobility and downward mobility models test the *independent* effect of social mobility on MoCA after accounting for the relative importance of childhood and adulthood SEP. 95% confidence intervals (CI) are given in brackets. AIC: Akaike Information Criteria. BIC: Bayesian Information Criteria. *** p<.001; ** p<.01; * p<.05.

**Table S4.** Logistic diagonal reference models (DRMs) estimating social mobility effects on the Mini Mental State Examination (MMSE) with additional adjustment for lifestyle and disease factors.

|  | ***Baseline model*** |  | ***Mobility***  ***(any direction) model*** |  | ***Upward mobility model*** | | ***Downward mobility model*** | | | |
| --- | --- | --- | --- | --- | --- | --- | --- | --- | --- | --- |
|  | OR | *95% CI* | OR | *95% CI* | OR | *95% CI* | | OR | | *95% CI* |
| Constant | 0.08^***^ | [0.02, 0.40] | 0.08^***^ | [0.02, 0.39] | 0.08^***^ | [0.02, 0.39] | | | 0.08^***^ | [0.01, 0.38] |
| **Socially stable groups** |  |  |  |  |  |  | | |  |  |
| Professional / managerial | 0.38^***^ | [0.27, 0.54] | 0.37^***^ | [0.26, 0.54] | 0.38^***^ | [0.27, 0.54] | | | 0.36^***^ | [0.23, 0.55] |
| Non-manual /skilled manual | 1.05 | [0.84, 1.32] | 1.06 | [0.84, 1.33] | 1.02 | [0.82, 1.27] | | | 1.10 | [0.81, 1.50] |
| Semi-skilled/unskilled/never worked | 2.51^***^ | [1.93, 3.25] | 2.55^***^ | [1.95, 3.33] | 2.55^***^ | [1.95, 3.32] | | | 2.55^***^ | [1.95, 3.33] |
| **Controls** |  |  |  |  |  |  | | |  |  |
| Female | 0.63^**^ | [0.45, 0.88] | 0.63^**^ | [0.45, 0.88] | 0.63^**^ | [0.45, 0.88] | | | 0.63^**^ | [0.45, 0.88] |
| Age | 1.09^***^ | [1.07, 1.11] | 1.09^***^ | [1.07, 1.11] | 1.09^***^ | [1.07, 1.11] | | | 1.09^***^ | [1.07, 1.11] |
| Childhood health | 1.27^***^ | [1.11, 1.45] | 1.26^***^ | [1.11, 1.44] | 1.26^***^ | [1.11, 1.44] | | | 1.26^***^ | [1.11, 1.44] |
| Current smoker | 1.23 | [0.78, 1.93] | 1.23 | [0.78, 1.93] | 1.23 | [0.78, 1.93] | | | 1.23 | [0.78, 1.93] |
| Excessive alcohol | 0.64 | [0.34, 1.24] | 0.64 | [0.34, 1.23] | 0.64 | [0.33, 1.23] | | | 0.65 | [0.34, 1.24] |
| Waist-Hip ratio | 1.00 | [0.99, 1.01] | 1.00 | [0.99, 1.01] | 1.00 | [0.99, 1.01] | | | 1.00 | [0.99, 1.01] |
| Chronic diseases | 1.30 | [0.94, 1.78] | 1.29 | [0.94, 1.78] | 1.29 | [0.94, 1.78] | | | 1.29 | [0.94, 1.78] |
| Cardiovascular diseases | 0.84 | [0.58, 1.22] | 0.84 | [0.58, 1.22] | 0.84 | [0.58, 1.22] | | | 0.85 | [0.59, 1.22] |
| Systolic blood pressure | 1.00 | [0.99, 1.01] | 1.00 | [0.99, 1.01] | 1.00 | [0.99, 1.01] | | | 1.00 | [0.99, 1.01] |
| Diastolic blood pressure | 0.99 | [0.97, 1.01] | 0.99 | [0.97, 1.01] | 0.99 | [0.97, 1.01] | | | 0.99 | [0.97, 1.01] |
| High levels of physical activity | 0.52^**^ | [0.34, 0.81] | 0.52^**^ | [0.34, 0.81] | 0.52^**^ | [0.34, 0.81] | | | 0.52^**^ | [0.34, 0.81] |
| Depression | 1.05^***^ | [1.02, 1.08] | 1.05^***^ | [1.01, 1.08] | 1.05^***^ | [1.01, 1.08] | | | 1.05^***^ | [1.01, 1.08] |
| Antidepressants | 1.77^***^ | [1.22, 2.58] | 1.77^***^ | [1.22, 2.58] | 1.78^***^ | [1.22, 2.59] | | | 1.77^***^ | [1.21, 2.57] |
| Antihypertensives | 0.87 | [0.60, 1.28] | 0.88 | [0.60, 1.28] | 0.87 | [0.60, 1.28] | | | 0.88 | [0.60, 1.28] |
| **Weight parameters** |  |  |  |  |  |  | | |  |  |
| Childhood | 0.08 | [-0.12, 0.29] | 0.09 | [-0.11, 0.29] | -0.01 | [-0.32, 0.29] | | | 0.20 | [-0.14, 0.54] |
| Adulthood | 0.92^***^ | [0.70, 1.12] | 0.91^***^ | [0.70, 1.10] | 1.01^***^ | [0.70, 1.31] | | | 0.80^***^ | [0.45, 1.15] |
| **Mobility status** |  |  |  |  |  |  | | |  |  |
| Mobile |  |  | 1.10 | [0.83, 1.47] |  |  | | |  |  |
| Upward |  |  |  |  | 1.22 | [0.71, 2.10] | | |  |  |
| Downward |  |  |  |  |  |  | | | 1.20 | [0.65, 2.23] |
| AIC | 1555 |  | 1556 |  | 1556 |  | | | 1556 |  |
| BIC | 1696 |  | 1705 |  | 1705 |  | | | 1705 |  |
| Degrees of freedom | 22 |  | 23 |  | 23 |  | | | 23 |  |
| Observations | 4620 |  | 4620 |  | 4620 |  | | | 4620 |  |

Note. *OR* = Odds Ratios. The *Constant* denotes average odds for a male of average age and average childhood health. *Socially stable groups*: OR are given for each socially stable group. They indicate the class specific deviations from the constant. *Controls*: all models are adjusted for age, sex, childhood health, cardiovascular diseases, chronic diseases, blood pressure, waist hip ratio, smoking status, alcohol consumption, physical activity, depression, antidepressants and antihypertensive medications. *Weight parameters*: bounded between 0 and 1, they express the relative contribution of childhood SEP and adulthood SEP on MMSE for the socially mobile. A high value of the childhood weight means that childhood class has greater influence on MMSE, while a high weight for adulthood means that adulthood class has greater influence on MMSE. The mobility (any direction), upward mobility and downward mobility models test the *independent* effect of social mobility on MMSE after accounting for the relative importance of childhood and adulthood SEP. 95% confidence intervals (CI) are given in brackets. AIC: Akaike Information Criteria. BIC: Bayesian Information Criteria. *** p<.001; ** p<.01; * p<.05.

**Table S5.** Point estimates from diagonal reference models (DRMs) estimating social mobility effects on the ventro-medial prefrontal cortex (vmPFC) volume with additional adjustment for lifestyle and disease factors.

|  | ***Baseline model*** |  | ***Mobility***  ***(any direction) model*** |  | ***Upward mobility model*** | | ***Downward mobility model*** | | | |
| --- | --- | --- | --- | --- | --- | --- | --- | --- | --- | --- |
|  | *b* | *95% CI* | *b* | *95% CI* | *b* | *95% CI* | | *b* | | *95% CI* |
| Constant | 10850^***^ | [9995, 11704] | 10855^***^ | [9998, 11711] | 10836^***^ | [9982, 11691] | | | 10889^***^ | [10033, 11744] |
| **Socially stable groups** |  |  |  |  |  |  | | |  |  |
| Professional / managerial | 143.2^*^ | [13.1, 273.3] | 141.7^*^ | [10.9, 272.6] | 154.5^*^ | [22.9, 286.1] | | | 145.8^*^ | [16.4, 275.3] |
| Non-manual /skilled manual | -62.4 | [-153.9, 29.0] | -62.5 | [-154.4, 29.3] | -56.8 | [-138.2, 24.4] | | | -55.2 | [-135.3, 24.8] |
| Semi-skilled/unskilled/never worked | -80.8 | [-191.2, 22.6] | -79.2 | [-190.9, 32.6] | -97.6 | [-204.6, 9.34] | | | -90.6 | [-189.3, 8.08] |
| **Controls** |  |  |  |  |  |  | | |  |  |
| Female | -606.3^***^ | [-803.9, -408.8] | -605.1^***^ | [-802.9, -407.1] | -613.9^***^ | [-811.8, -416.2] | | | -601.2^***^ | [-798.4, -404.1] |
| Age | -43.5^***^ | [-54.5, -32.4] | -43.5^***^ | [-54.5, -32.4] | -43.5^***^ | [-54.5, -32.5] | | | -43.3^***^ | [-54.3, -32.3] |
| eTIV | 0.00^***^ | [0.00, 0.00] | 0.0^***^ | [0.00, 0.00] | 0.0^***^ | [0.00, 0.00] | | | 0.0^***^ | [0.00, 0.00] |
| Childhood health | 1.48 | [-76.4, 79.4] | 1.73 | [-76.2, 79.7] | 1.09 | [-76.7, 78.9] | | | 3.80 | [-74.0, 81.6] |
| Current smoker | -167.5 | [-478.7, 143.7] | -166.0 | [-478.2, 144.3] | -163.8 | [-474.8, 147.1] | | | -154.2 | [-380.9, 72.5] |
| Excessive alcohol | -216.1 | [-467.9, 35.8] | -217.2 | [-469.3, 34.9] | -213.6 | [-465.2, 37.9] | | | -225.2 | [-476.9, 26.5] |
| Waist-Hip ratio | 2.37 | [-3.6, 8.4] | 2.41 | [-3.6, 8.4] | 2.14 | [-3.9, 8.2] | | | 2.41 | [-3.6, 8.4] |
| Chronic diseases | -34.8 | [-181.8, 112.1] | -34.6 | [-181.6, 112.3] | -35.8 | [-182.6, 110.9] | | | -33.4 | [-180.0, 113.3] |
| Cardiovascular diseases | -104.7 | [-320.6, 111.2] | -104.9 | [-320.9, 110.9] | -98.5 | [-314.8, 117.7] | | | -98.2 | [-314.2, 117.8] |
| Systolic blood pressure | 2.32 | [-3.6, 8.3] | 2.33 | [-3.6, 8.3] | 2.19 | [-3.7, 8.2] | | | 2.15 | [-3.8, 8.1] |
| Diastolic blood pressure | -9.6 | [-20.1, 0.89] | -9.6 | [-20.1, 0.89] | -9.4 | [-19.8, 1.13] | | | -9.3 | [-19.8, 1.15] |
| High levels of physical activity | 23.1 | [-162.9, 209.2] | 23.3 | [-162.8, 209.3] | 22.4 | [-163.5, 208.3] | | | 23.9 | [-161.7, 209.7] |
| Depression | -8.4 | [-29.7, 12.8] | -8.6 | [-29.9, 12.8] | -7.5 | [-28.9, 13.8] | | | -8.6 | [-29.9, 12.7] |
| Antidepressants | -111.3 | [-393.6, 170.9] | -111.0 | [-393.3, 171.2] | -113.1 | [-395.1, 168.8] | | | -110.0 | [-391.7, 171.7] |
| Antihypertensives | 31.6 | [-185.6, 248.9] | 30.7 | [-186.8, 248.3] | 30.8 | [-186.4, 248.1] | | | 19.4 | [-198.5, 237.3] |
| **Weight parameters** |  |  |  |  |  |  | | |  |  |
| Childhood | 1.07^**^ | [0.32, 1.82] | 1.07^**^ | [0.31, 1.82] | 1.30^**^ | [0.32, 2.29] | | | 1.35^**^ | [0.32, 2.37] |
| Adulthood | -0.07 | [-0.82, 0.67] | -0.07 | [-0.82, 0.68] | -0.30 | [-1.29, 0.68] | | | -0.35 | [-1.37, 0.67] |
| **Mobility status** |  |  |  |  |  |  | | |  |  |
| Mobile |  |  | -13.5 | [-160.8, 133.8] |  |  | | |  |  |
| Upward |  |  |  |  | 101.6 | [-137.2, 340.5] | | |  |  |
| Downward |  |  |  |  |  |  | | | -153.8 | [-404.8, 97.1] |
| AIC | 7509 |  | 7509 |  | 7508 |  | | | 7509 |  |
| BIC | 7608 |  | 7608 |  | 7607 |  | | | 7613 |  |
| Degrees of freedom | 24 |  | 25 |  | 25 |  | | | 25 |  |
| Observations | 464 |  | 464 |  | 464 |  | | | 464 |  |

Note. The *Constant* denotes average vmPFC volume for a male of average age, average childhood health and average estimated intracranial volume (eTIV). *Socially stable groups*: the class coefficients indicate the class specific deviations from the constant. *Controls*: all models are adjusted for age, sex, eTIV, childhood health, cardiovascular diseases, chronic diseases, blood pressure, waist hip ratio, smoking status, alcohol consumption, physical activity, depression, antidepressants and antihypertensive medications. *Weight parameters*: bounded between 0 and 1, they express the relative contribution of childhood SEP and adulthood SEP on the vmPFC volume for the socially mobile. A high value of the childhood weight means that childhood class has greater influence on the vmPFC, while a high weight for adulthood means that adulthood class has greater influence on the vmPFC. The mobility (any direction), upward mobility and downward mobility models test the *independent* effect of social mobility on the vmPFC volume after accounting for the relative importance of childhood and adulthood SEP. 95% confidence intervals (CI) are given in brackets. AIC: Akaike Information Criteria. BIC: Bayesian Information Criteria. *** p<.001; ** p<.01; * p<.05.

**Table S6.** Point estimates from diagonal reference models (DRMs) estimating social mobility effects on the anterior cingulate (AC) volume with additional adjustment for lifestyle and disease factors.

|  | ***Baseline model*** |  | ***Mobility***  ***(any direction) model*** |  | ***Upward mobility model*** | | ***Downward mobility model*** | | | |
| --- | --- | --- | --- | --- | --- | --- | --- | --- | --- | --- |
|  | *b* | *95% CI* | *b* | *95% CI* | *b* | *95% CI* | | *b* | | *95% CI* |
| Constant | 7823^***^ | [6602, 9044] | 7842^***^ | [6619, 9065] | 7820^***^ | [6599, 9042] | | | 7891^***^ | [6669, 9114] |
| **Socially stable groups** |  |  |  |  |  |  | | |  |  |
| Professional / managerial | 201.6^*^ | [26.2, 377.1] | 203.8^*^ | [30.5, 377.3] | 184.7^*^ | [-6.0, 375.5] | | | 157.5 | [-33.3, 348.3] |
| Non-manual /skilled manual | 230.8^**^ | [37.6, 424.1] | 224.8^*^ | [29.5, 420.1] | 245.8^**^ | [48.5, 443.1] | | | 253.0^**^ | [61.9, 444.2] |
| Semi-skilled/unskilled/never worked | -432.5^***^ | [-649.6, -215.4] | -428.7^***^ | [-646.6, -210.7] | -430.5^***^ | [-647.6, -213.5] | | | -410.6^***^ | [-629.7, -191.4] |
| **Controls** |  |  |  |  |  |  | | |  |  |
| Female | -89.7 | [-372.7, 193.3] | -83.6 | [-367.4, 200.1] | -93.4 | [-376.6, 189.8] | | | -77.4 | [-360.3, 205.5] |
| Age | -35.5^***^ | [-51.2, -19.8] | -35.4^***^ | [-51.1, -19.6] | -35.8^***^ | [-51.6, -20.1] | | | -36.1^***^ | [-51.8, -20.4] |
| eTIV | 0.00^***^ | [0.00, 0.00] | 0.00^***^ | [0.00, 0.00] | 0.00^***^ | [0.00, 0.00] | | | 0.00^***^ | [0.00, 0.00] |
| Childhood health | -33.8 | [-145.2, 77.6] | -32.7 | [-144.2, 78.7] | -34.3 | [-145.7, 77.1] | | | -30.8 | [-142.2, 80.4] |
| Current smoker | 31.6 | [-417.6, 480.8] | 36.6 | [-412.8, 486.1] | 23.4 | [-427.1, 473.9] | | | 25.6 | [-423.2, 474.4] |
| Excessive alcohol | -400.0^*^ | [-760.7, -39.3] | -400.3^*^ | [-765.3, -43.3] | -399.1^*^ | [-759.7, -38.4] | | | -414.5^*^ | [-775.0, -54.0] |
| Waist-Hip ratio | 1.5 | [-7.18, 10.2] | 1.6 | [-7.04, 10.4] | 1.3 | [-7.3, 10.1] | | | 1.6 | [-7.0, 10.3] |
| Chronic diseases | 209.7^*^ | [-154, 420.9] | 210.7^*^ | [-0.54, 422.1] | 207.9^*^ | [-3.2, 419.1] | | | 207.8^*^ | [-2.6, 418.2] |
| Cardiovascular diseases | 159.3 | [-154.6, 473.3] | 159.1 | [-154.7, 472.9] | 157.4 | [-156.7, 471.5] | | | 152.2 | [-161.6, 466.0] |
| Systolic blood pressure | 3.1 | [-5.4, 11.6] | 3.1 | [-5.4, 11.6] | 2.9 | [-5.6, 11.5] | | | 2.8 | [-5.7, 11.4] |
| Diastolic blood pressure | -4.1 | [-19.2, 10.9] | -4.1 | [-19.2, 10.9] | -4.0 | [-19.1, 11.1] | | | -3.9 | [-19.0, 11.1] |
| High levels of physical activity | -14.8 | [-280.7, 250.9] | -14.9 | [-280.7, 250.9] | -12.5 | [-278.5, 253.4] | | | -7.2 | [-390.4, 84.4] |
| Depression | -3.9 | [-34.5, 26.6] | -4.5 | [-35.1, 26.2] | -3.4 | [-34.1, 27.2] | | | -4.3 | [-34.8, 26.2] |
| Antidepressants | -148.1 | [-551.5, 255.4] | -147.0 | [-550.5, 256.4] | -146.1 | [-549.3, 257.2] | | | -136.6 | [-539.2, 265.9] |
| Antihypertensives | -131.2 | [-443.2, 180.7] | -136.7 | [-449.3, 175.8] | -124.6 | [-437.7, 188.5] | | | -131.8 | [-443.4, 179.7] |
| **Weight parameters** |  |  |  |  |  |  | | |  |  |
| Childhood | 0.28 | [-0.01, 0.57] | 0.25 | [-0.05, 0.57] | 0.35 | [-0.03, 0.72] | | | 0.42^*^ | [0.08, 0.75] |
| Adulthood | 0.72^***^ | [0.42, 1.01] | 0.75^***^ | [0.42, 1.05] | 0.65^***^ | [0.28, 1.02] | | | 0.58^***^ | [0.25, 0.91] |
| **Mobility status** |  |  |  |  |  |  | | |  |  |
| Mobile |  |  | -56.7 | [-265.8, 152.4] |  |  | | |  |  |
| Upward |  |  |  |  | 70.3 | [-202.4, 343.1] | | |  |  |
| Downward |  |  |  |  |  |  | | | -229.8 | [-539.6, 79.9] |
| AIC | 7841 |  | 7842 |  | 7842 |  | | | 7841 |  |
| BIC | 7940 |  | 7946 |  | 7946 |  | | | 7944 |  |
| Degrees of freedom | 24 |  | 25 |  | 25 |  | | | 25 |  |
| Observations | 464 |  | 464 |  | 464 |  | | | 464 |  |

Note. The *Constant* denotes average AC volume for a male of average age, average childhood health and average estimated intracranial volume (eTIV). *Socially stable groups*: the class coefficients indicate the class specific deviations from the constant. *Controls*: all models are adjusted for age, sex, eTIV, childhood health, cardiovascular diseases, chronic diseases, blood pressure, waist hip ratio, smoking status, alcohol consumption, physical activity, depression, antidepressants and antihypertensive medications. *Weight parameters*: bounded between 0 and 1, they express the relative contribution of childhood SEP and adulthood SEP on the AC volume for the socially mobile. A high value of the childhood weight means that childhood class has greater influence on the AC, while a high weight for adulthood means that adulthood class has greater influence on the AC. The mobility (any direction), upward mobility and downward mobility models test the *independent* effect of social mobility on the AC volume after accounting for the relative importance of childhood and adulthood SEP. 95% confidence intervals (CI) are given in brackets. AIC: Akaike Information Criteria. BIC: Bayesian Information Criteria. *** p<.001; ** p<.01; * p<.05.

**Table S7.** Point estimates from diagonal reference models (DRMs) estimating social mobility effects on total grey matter volume (GMV) with additional adjustment for lifestyle and disease factors.

|  | ***Baseline model*** |  | ***Mobility***  ***(any direction) model*** |  | ***Upward mobility model*** | | ***Downward mobility model*** | | | | |
| --- | --- | --- | --- | --- | --- | --- | --- | --- | --- | --- | --- |
|  | *b* | *95% CI* | *b* | *95% CI* | *b* | *95% CI* | | *b* | | *95% CI* | |
| Constant | 625446^***^ | [594963, 655929] | 625414^***^ | [594839, 655990] | 625611^***^ | [595114, 656108] | | | 625244^***^ | [594735, 655753] |  |
| **Socially stable groups** |  |  |  |  |  |  | | |  |  | |
| Professional / managerial | 2236.9 | [-28530.8, -14376.2] | 2246.9 | [-2864.7, 7358.7] | 2144.0 | [-2585.3, 6873.3] | | | 2210.3 | [-2485.2, 6905.9] | |
| Non-manual /skilled manual | 2512.2 | [-2658.3, 7682.9] | 2506.3 | [-2716.8, 7729.5] | 2620.5 | [-2062.4, 7303.5] | | | 2632.8 | [-2048.0, 7313.6] | |
| Semi-skilled/unskilled/never worked | -4749.2 | [-10153.4, 655.0] | -4753.3 | [-10166.0, 659.3] | -4764.5 | [-10189.8, 660.6] | | | -4843.1 | [-10304.8, 618.5] | |
| **Controls** |  |  |  |  |  |  | | |  |  | |
| Female | -21453.5^***^ | [-803.9, -408.8] | -21459.4^***^ | [-28551.3, -14367.5] | -21443.2^***^ | [-28512.1, -14374.2] | | | -21535.3^***^ | [-28622.8, -14447.8] | |
| Age | -3004.4^***^ | [-3397.1, -2611.6] | -3004.7^***^ | [-3398.0, -2611.3] | -2998.9^***^ | [-3394.6, -2603.2] | | | -2999.6^***^ | [-3393.3, -2605.8] | |
| eTIV | 0.16^***^ | [0.14, 0.18] | 0.16^***^ | [0.14, 0.18] | 0.16^***^ | [0.14, 0.18] | | | 0.16^***^ | [0.14, 0.18] | |
| Childhood health | -1012.6 | [-3787.1, 1761.9] | -1013.8 | [-3789.7, 1762.1] | -1012.0 | [-3786.7, 1762.6] | | | -1030.0 | [-3807.3, 1747.2] | |
| Current smoker | -13911.4^**^ | [-25085.6, -2737.3] | -13916.6^**^ | [-25097.4, -2735.7] | -13864.5^**^ | [-25050.4, -2678.5] | | | -13914.9^**^ | [-25090.5, -2739.4] | |
| Excessive alcohol | -12041.2^**^ | [-21020.3, -3062.0] | -12035.6^**^ | [-11361.5, 4817.3] | -12052.2^**^ | [-21031.0, -3073.4] | | | -11975.7^*^ | [-20966.8, -2984.6] | |
| Waist-Hip ratio | -31.3 | [-250.8, 188.2] | -31.3 | [-251.0, 188.2] | -31.5 | [-249.9, 186.8] | | | -33.3 | [-252.1, 185.3] | |
| Chronic diseases | 2845.5 | [-2400.7, 8091.8] | 2843.0 | [-2406.5, 8092.7] | 2887.8 | [-2372.5, 8148.2] | | | 2877.0 | [-2372.9, 8127.0] | |
| Cardiovascular diseases | -4793.6 | [-12779.5, 3192.2] | -4797.5 | [-12792.5, 3197.5] | -4713.8 | [-12659.6, 3231.8] | | | -4709.1 | [-12645.7, 3227.4] | |
| Systolic blood pressure | 113.8 | [-100.7, 328.4] | 113.9 | [-100.7, 328.5] | 113.8 | [-100.5, 328.1] | | | 114.2 | [-100.1, 328.6] | |
| Diastolic blood pressure | -468.5^*^ | [-846.3, -90.8] | -468.6^*^ | [-846.5, -90.7] | -467.6^*^ | [-844.7, -90.5] | | | -467.8^*^ | [-844.9, -90.7] | |
| High levels of physical activity | -4229.7 | [-10856.1, 2396.7] | -4229.5 | [-10856.0, 2396.8] | -4254.3 | [-10885.1, 2376.4] | | | -4268.12 | [-10900.0, 2363.8] | |
| Depression | 152.4 | [-615.3, 920.2] | 153.1 | [-616.6, 923.0] | 145.6 | [-625.0, 916.3] | | | 153.4 | [-614.0, 921.0] | |
| Antidepressants | -8299.0 | [-18357.0, 1758.9] | -8297.2 | [-18356.1, 1761.5] | -8371.3 | [-18448.1, 1705.4] | | | -8393.1 | [-18468.5, 1682.3] | |
| Antihypertensives | 1292.3 | [-6543.2, 9127.8] | 1301.3 | [-6565.1, 9167.9] | 1195.0 | [-6667.1, 9057.2] | | | 1266.9 | [-6548.8, 9082.8] | |
| **Weight parameters** |  |  |  |  |  |  | | |  |  | |
| Childhood | 0.33 | [-0.42, 1.08] | 0.33 | [-0.45, 1.11] | 0.27 | [-0.60, 1.13] | | | 0.27 | [-0.51, 1.04] | |
| Adulthood | 0.67 | [-0.08, 1.42] | 0.67 | [-0.11, 1.45] | 0.73 | [-0.13, 1.60] | | | 0.73 | [-0.04, 1.51] | |
| **Mobility status** |  |  |  |  |  |  | | |  |  | |
| Mobile |  |  | 69.2 | [-5162.7, 5301.2] |  |  | | |  |  | |
| Upward |  |  |  |  | -667.8 | [-7238.0, 5902.3] | | |  |  | |
| Downward |  |  |  |  |  |  | | | 1076.4 | [-6762.1, 8915.1] | |
| AIC | 10824 |  | 10826 |  | 10826 |  | | | 10826 |  | |
| BIC | 10923 |  | 10929 |  | 10929 |  | | | 10929 |  | |
| Degrees of freedom | 24 |  | 25 |  | 25 |  | | | 25 |  | |
| Observations | 464 |  | 464 |  | 464 |  | | | 464 |  | |

Note. The *Constant* denotes average total GMV for a male of average age, average childhood health and average estimated intracranial volume (eTIV). *Socially stable groups*: the class coefficients indicate the class specific deviations from the constant. *Controls*: all models are adjusted for age, sex, eTIV, childhood health, cardiovascular diseases, chronic diseases, blood pressure, waist hip ratio, smoking status, alcohol consumption, physical activity, depression, antidepressants and antihypertensive medications. *Weight parameters*: bounded between 0 and 1, they express the relative contribution of childhood SEP and adulthood SEP on total GMV for the socially mobile. A high value of the childhood weight means that childhood class has greater influence on GMV, while a high weight for adulthood means that adulthood class has greater influence on GMV. The mobility (any direction), upward mobility and downward mobility models test the *independent* effect of social mobility on total GMV after accounting for the relative importance of childhood and adulthood SEP. 95% confidence intervals (CI) are given in brackets. AIC: Akaike Information Criteria. BIC: Bayesian Information Criteria. *** p<.001; ** p<.01; * p<.05.

**Table S8.** Logistic diagonal reference models (DRMs) estimating social mobility effects on the Montreal Cognitive Assessment (MoCA) with additional adjustment for lifestyle and disease factors AND performance on the National Adult Reading Test (NART).

|  | ***Baseline model*** |  | ***Mobility***  ***(any direction) model*** |  | ***Upward mobility model*** | | ***Downward mobility model*** | | | |
| --- | --- | --- | --- | --- | --- | --- | --- | --- | --- | --- |
|  | OR | *95% CI* | OR | *95% CI* | OR | *95% CI* | | OR | | *95% CI* |
| Constant | 0.01^***^ | [0.00, 0.05] | 0.01^***^ | [0.00, 0.05] | 0.01^***^ | [0.00, 0.05] | | | 0.01^***^ | [0.00, 0.06] |
| **Socially stable groups** |  |  |  |  |  |  | | |  |  |
| Professional / managerial | 0.96 | [0.80,1.14] | 0.96 | [0.81,1.14] | 0.94 | [0.77,1.14] | | | 0.94 | [0.80,1.11] |
| Non-manual /skilled manual | 0.94 | [0.80,1.09] | 0.94 | [0.80,1.10] | 0.94 | [0.82,1.08] | | | 0.95 | [0.82,1.08] |
| Semi-skilled/unskilled/never worked | 1.11 | [0.85,1.45] | 1.11 | [0.85,1.45] | 1.13 | [0.86,1.49] | | | 1.12 | [0.86,1.46] |
| **Controls** |  |  |  |  |  |  | | |  |  |
| Female | 0.86 | [0.62,1.21] | 0.86 | [0.62,1.21] | 0.86 | [0.62,1.20] | | | 0.87 | [0.62,1.21] |
| Age | 1.15^***^ | [1.12,1.17] | 1.15^***^ | [1.13,1.17] | 1.15^***^ | [1.12,1.17] | | | 1.15^***^ | [1.13,1.17] |
| Childhood health | 0.96 | [0.83,1.11] | 0.96 | [0.83,1.11] | 0.96 | [0.83,1.11] | | | 0.96 | [0.84,1.11] |
| Current smoker | 1.19 | [0.78,1.84] | 1.20 | [0.78,1.84] | 1.19 | [0.77,1.83] | | | 1.20 | [0.78,1.85] |
| Excessive alcohol | 1.07 | [0.60,1.92] | 1.08 | [0.60,1.93] | 1.07 | [0.60,1.92] | | | 1.08 | [0.60,1.93] |
| Waist-Hip ratio | 0.99 | [0.98, 1.00] | 0.99 | [0.98, 1.00] | 0.99 | [0.98, 1.00] | | | 0.99 | [0.98, 1.00] |
| Chronic diseases | 0.96 | [0.71, 1.29] | 0.96 | [0.71, 1.29] | 0.95 | [0.71, 1.29] | | | 0.95 | [0.71, 1.29] |
| Cardiovascular diseases | 1.06 | [0.74,1.52] | 1.06 | [0.74,1.52] | 1.06 | [0.74,1.51] | | | 1.05 | [0.74,1.51] |
| Systolic blood pressure | 0.99^*^ | [0.98, 1.00] | 0.99^*^ | [0.98, 1.00] | 0.99^*^ | [0.98, 1.00] | | | 0.99^*^ | [0.98, 1.00] |
| Diastolic blood pressure | 1.01 | [0.99, 1.03] | 1.01 | [0.99, 1.03] | 1.01 | [0.99, 1.03] | | | 1.01 | [0.99, 1.03] |
| High levels of physical activity | 0.59^*^ | [0.39,0.88] | 0.59^*^ | [0.39,0.88] | 0.59^*^ | [0.39,0.88] | | | 0.59^*^ | [0.39,0.88] |
| Depression | 1.06^***^ | [1.03, 1.09] | 1.06^***^ | [1.03, 1.09] | 1.06^***^ | [1.03, 1.09] | | | 1.06^***^ | [1.02, 1.09] |
| Antidepressants | 1.98^***^ | [1.35, 2.90] | 1.98^***^ | [1.35, 2.90] | 1.99^***^ | [1.36, 2.91] | | | 1.99^***^ | [1.35, 2.90] |
| Antihypertensives | 0.77 | [0.53, 1.11] | 0.77 | [0.53, 1.11] | 0.77 | [0.53, 1.11] | | | 0.77 | [0.53, 1.11] |
| NART error score | 1.13^***^ | [1.11, 1.15] | 1.13^***^ | [1.11, 1.15] | 1.13^***^ | [1.11, 1.15] | | | 1.13^***^ | [1.11, 1.15] |
| **Weight parameters** |  |  |  |  |  |  | | |  |  |
| Childhood | -0.95 | [-4.53, 2.63] | -0.97 | [-4.68, 2.73] | -1.14 | [-4.83, 2.54] | | | -1.48 | [-6.24, 3.27] |
| Adulthood | 1.95 | [-1.63, 5.53] | 1.97 | [-1.73, 5.68] | 2.14 | [-1.54, 5.83] | | | 2.48 | [-2.27, 7.24] |
| **Mobility status** |  |  |  |  |  |  | | |  |  |
| Mobile |  |  | 0.97 | [0.73, 1.28] |  |  | | |  |  |
| Upward |  |  |  |  | 1.13 | [0.68,1.85] | | |  |  |
| Downward |  |  |  |  |  |  | | | 0.81 | [0.49,1.32] |
| AIC | 1516 |  | 1518 |  | 1518 |  | | | 1517 |  |
| BIC | 1664 |  | 1672 |  | 1672 |  | | | 1672 |  |
| Degrees of freedom | 23 |  | 24 |  | 24 |  | | | 24 |  |
| Observations | 4620 |  | 4620 |  | 4620 |  | | | 4620 |  |

Note. *OR* = Odds Ratios. The *Constant* denotes average odds for a male of average age and average childhood health. *Socially stable groups*: OR are given for each socially stable group. They indicate the class specific deviations from the constant. *Controls*: all models are adjusted for age, sex, childhood health, cardiovascular diseases, chronic diseases, blood pressure, waist hip ratio, smoking status, alcohol consumption, physical activity, depression, antidepressants, antihypertensive medications and NART error score. *Weight* *parameters*: bounded between 0 and 1, they express the relative contribution of childhood SEP and adulthood SEP on MoCA for the socially mobile. A high value of the childhood weight means that childhood class has greater influence on MoCA, while a high weight for adulthood means that adulthood class has greater influence on MoCA. The mobility (any direction), upward mobility and downward mobility models test the *independent* effect of social mobility on MoCA after accounting for the relative importance of childhood and adulthood SEP. 95% confidence intervals (CI) are given in brackets. AIC: Akaike Information Criteria. BIC: Bayesian Information Criteria. *** p<.001; ** p<.01; * p<.05.

**Table S9.** Logistic diagonal reference models (DRMs) estimating social mobility effects on the Mini Mental State Examination (MMSE) with additional adjustment for lifestyle and disease factors AND performance on the National Adult Reading Test (NART).

|  | ***Baseline model*** |  | ***Mobility***  ***(any direction) model*** |  | ***Upward mobility model*** | | ***Downward mobility model*** | | | |
| --- | --- | --- | --- | --- | --- | --- | --- | --- | --- | --- |
|  | OR | *95% CI* | OR | *95% CI* | OR | *95% CI* | | OR | | *95% CI* |
| Constant | 0.01^***^ | [0.00, 0.04] | 0.01^***^ | [0.00, 0.04] | 0.01^***^ | [0.00, 0.04] | | | 0.01^***^ | [0.00, 0.04] |
| **Socially stable groups** |  |  |  |  |  |  | | |  |  |
| Professional / managerial | 0.90 | [0.71,1.14] | 0.89 | [0.69,1.15] | 0.87 | [0.68,1.12] | | | 0.90 | [0.71,1.14] |
| Non-manual /skilled manual | 0.95 | [0.86,1.05] | 0.95 | [0.86,1.05] | 0.95 | [0.87,1.04] | | | 0.95 | [0.86,1.05] |
| Semi-skilled/unskilled/never worked | 1.17 | [0.86,1.58] | 1.18 | [0.87,1.61] | 1.21 | [0.89,1.64] | | | 1.17 | [0.86,1.58] |
| **Controls** |  |  |  |  |  |  | | |  |  |
| Female | 0.70^*^ | [0.49,1.00] | 0.69^*^ | [0.49,0.99] | 0.69^*^ | [0.48,0.99] | | | 0.70^*^ | [0.49, 1.00] |
| Age | 1.09^***^ | [1.07, 1.11] | 1.09^***^ | [1.07, 1.11] | 1.09^***^ | [1.07, 1.11] | | | 1.09^***^ | [1.07, 1.11] |
| Childhood health | 1.23^**^ | [1.07, 1.42] | 1.23^**^ | [1.07, 1.42] | 1.23^**^ | [1.07, 1.42] | | | 1.23^**^ | [1.07, 1.42] |
| Current smoker | 0.99 | [0.62,1.60] | 0.99 | [0.61,1.58] | 0.98 | [0.61,1.57] | | | 0.99 | [0.62,1.60] |
| Excessive alcohol | 0.67 | [0.34, 1.33] | 0.67 | [0.34, 1.32] | 0.67 | [0.34, 1.32] | | | 0.67 | [0.34, 1.33] |
| Waist-Hip ratio | 1.00 | [0.98, 1.01] | 1.00 | [0.98, 1.01] | 1.00 | [0.98, 1.01] | | | 1.00 | [0.98, 1.01] |
| Chronic diseases | 1.33 | [0.96, 1.85] | 1.33 | [0.95, 1.85] | 1.32 | [0.95, 1.84] | | | 1.33 | [0.96, 1.85] |
| Cardiovascular diseases | 0.75 | [0.51, 1.09] | 0.75 | [0.51, 1.09] | 0.74 | [0.51, 1.09] | | | 0.75 | [0.51, 1.09] |
| Systolic blood pressure | 1.00 | [0.99, 1.01] | 1.00 | [0.99, 1.01] | 1.00 | [0.99, 1.01] | | | 1.00 | [0.99, 1.01] |
| Diastolic blood pressure | 0.99 | [0.97, 1.02] | 0.99 | [0.97, 1.02] | 0.99 | [0.97, 1.02] | | | 0.99 | [0.97, 1.02] |
| High levels of physical activity | 0.52^**^ | [0.33, 0.81] | 0.51^**^ | [0.33, 0.81] | 0.51^**^ | [0.33, 0.81] | | | 0.52^**^ | [0.33, 0.81] |
| Depression | 1.05^**^ | [1.01, 1.08] | 1.05^**^ | [1.01, 1.08] | 1.05^**^ | [1.01, 1.08] | | | 1.05^**^ | [1.01, 1.08] |
| Antidepressants | 1.77^**^ | [1.19, 2.64] | 1.78^**^ | [1.19, 2.65] | 1.78^**^ | [1.20, 2.66] | | | 1.77^**^ | [1.19, 2.64] |
| Antihypertensives | 0.87 | [0.59, 1.29] | 0.87 | [0.59, 1.29] | 0.87 | [0.59, 1.29] | | | 0.87 | [0.59, 1.28] |
| NART error score | 1.11^***^ | [1.09,1.13] | 1.11^***^ | [1.09,1.13] | 1.11^***^ | [1.09,1.13] | | | 1.11^***^ | [1.09,1.13] |
| **Weight parameters** |  |  |  |  |  |  | | |  |  |
| Childhood | -1.73 | [-6.18, 2.71] | -1.56 | [-5.45, 2.33] | -1.94 | [-5.89, 1.99] | | | -1.80 | [-6.63, 3.02] |
| Adulthood | 2.73 | [-1.71, 7.18] | 2.56 | [-1.33, 6.45] | 2.94 | [-0.99, 6.89] | | | 2.80 | [-2.02, 7.63] |
| **Mobility status** |  |  |  |  |  |  | | |  |  |
| Mobile |  |  | 1.09 | [0.81, 1.47] |  |  | | |  |  |
| Upward |  |  |  |  | 1.36 | [0.71, 2.10] | | |  |  |
| Downward |  |  |  |  |  |  | | | 0.97 | [0.57, 1.65] |
| AIC | 1381 |  | 1383 |  | 1382 |  | | | 1383 |  |
| BIC | 1529 |  | 1537 |  | 1536 |  | | | 1537 |  |
| Degrees of freedom | 23 |  | 24 |  | 24 |  | | | 24 |  |
| Observations | 4620 |  | 4620 |  | 4620 |  | | | 4620 |  |

Note. *OR* = Odds Ratios. The *Constant* denotes average odds for a male of average age and average childhood health. *Socially stable groups*: OR are given for each socially stable group. They indicate the class specific deviations from the constant. *Controls*: all models are adjusted for age, sex, childhood health, cardiovascular diseases, chronic diseases, blood pressure, waist hip ratio, smoking status, alcohol consumption, physical activity, depression, antidepressants, antihypertensive medications and NART error score. Weight parameters: bounded between 0 and 1, they express the relative contribution of childhood SEP and adulthood SEP on MMSE for the socially mobile. A high value of the childhood weight means that childhood class has greater influence on MMSE, while a high weight for adulthood means that adulthood class has greater influence on MMSE. The mobility (any direction), upward mobility and downward mobility models test the *independent* effect of social mobility on MMSE after accounting for the relative importance of childhood and adulthood SEP. 95% confidence intervals are given in brackets. AIC: Akaike Information Criteria. BIC: Bayesian Information Criteria. *** p<.001; ** p<.01; * p<.05.

**Table S10.** Point estimates from diagonal reference models (DRMs) estimating social mobility effects on total grey matter volume (GMV) with additional adjustment for lifestyle and disease factors AND performance on the National Adult Reading Test (NART).

|  | ***Baseline model*** |  | ***Mobility***  ***(any direction) model*** |  | ***Upward mobility model*** | | ***Downward mobility model*** | | | | |
| --- | --- | --- | --- | --- | --- | --- | --- | --- | --- | --- | --- |
|  | *b* | *95% CI* | *b* | *95% CI* | *b* | *95% CI* | | *b* | | *95% CI* | |
| Constant | 626631^***^ | [595685, 657578] | 626661^***^ | [595685, 657578] | 626874^***^ | [595978, 657771] | | | 626407^***^ | [594735, 655753] |  |
| **Socially stable groups** |  |  |  |  |  |  | | |  |  | |
| Professional / managerial | 1529.6 | [-4323.0, 7382.3] | 1520.3 | [-4379.4, 7420.0] | 1451.8 | [-3854.0, 6757.8] | | | 1554.4 | [-3833.7, 6942.5] | |
| Non-manual /skilled manual | 2673.9 | [-2053.5, 7401.5] | 2675.9 | [-2037.1, 7389.0] | 2708.9 | [-1652.2, 7070.1] | | | 2720.6 | [-1702.6, 7143.9] | |
| Semi-skilled/unskilled/never worked | -4203.6 | [-10279.1, 1871.7] | -4196.2 | [-10315.6, 1923.1] | -4160.8 | [-10259.0, 1937.3] | | | -4275.0 | [-10369.6, 1819.4] | |
| **Controls** |  |  |  |  |  |  | | |  |  | |
| Female | -21606.3^***^ | [-28706.2, -14506.3] | -21601.6^***^ | [-28714.0, -14489.3] | -21584.7^***^ | [-28672.5, -14496.8] | | | -21680.2^***^ | [-28780.4, -14579.9] | |
| Age | -3008.4^***^ | [-3401.8, -2615.0] | -3008.2^***^ | [-3402.084, -2614.4] | -3002.5^***^ | [-3397.7, -2607.2] | | | -3004.0^***^ | [-3398.1, -2609.9] | |
| eTIV | 0.16^***^ | [0.14, 0.18] | 0.16^***^ | [0.14, 0.18] | 0.16^***^ | [0.14, 0.18] | | | 0.16^***^ | [0.14, 0.18] | |
| Childhood health | -950.9 | [-3737.9, 1835.9] | -949.7 | [-3738.91, 1839.3] | -946.4 | [-3733.1, 1840.2] | | | -968.0 | [-3757.0, 1820.8] | |
| Current smoker | -13998.5^**^ | [-25180.6, -2816.3] | -13995.1^**^ | [-25181.7, -2808.6] | -13953.0^**^ | [-25145.1, -2760.9] | | | -14009.6^**^ | [-25193.0, -2826.1] | |
| Excessive alcohol | -12084.8^**^ | [-21063.7, -3105.9] | -12088.9^**^ | [-21076.2, -3101.7] | -13953.0^**^ | [-21070.2, -3111.4] | | | -12009.5^*^ | [-21001.7, -3017.3] | |
| Waist-Hip ratio | -29.7 | [-248.4, 188.9] | -29.7 | [-248.5, 189.2] | -28.8 | [-247.0, 189.3] | | | -31.0 | [-249.2, 187.2] | |
| Chronic diseases | 2934.6 | [-2324.2, 8193.4] | 2936.9 | [-2326.2, 8200.1] | 2991.9 | [-2280.0, 8264.0] | | | 2968.5 | [-2293.4, 8230.4] | |
| Cardiovascular diseases | -4473.8 | [-12539.0, 3591.3] | -4402.9 | [-12537.9, 3596.7] | -4402.9 | [-12390.5, 3584.5] | | | -4419.2 | [-12413.7, 3575.1] | |
| Systolic blood pressure | 114.3 | [-100.9, 328.7] | 114.3 | [-100.1, 328.7] | 114.8 | [-99.3, 329.0] | | | 115.1 | [-99.1, 329.4] | |
| Diastolic blood pressure | -470.5^*^ | [-847.7, -93.3] | -470.5^*^ | [-847.7, -93.3] | -470.6^*^ | [-847.7, -93.3] | | | -470.6^*^ | [-847.6, -93.7] | |
| High levels of physical activity | -4168.2 | [-10800.1, 2463.7] | -4168.0 | [-10800.0, 2463.7] | -4194.7 | [-10829.1, 2439.5] | | | -4206.5 | [-10842.6, 2429.6] | |
| Depression | 156.3 | [-610.7, 923.3] | 155.6 | [-613.7, 924.9] | 146.2 | [-624.2, 916.6] | | | 156.0 | [-610.9, 922.9] | |
| Antidepressants | -8388.2 | [-18458.9, 1682.6] | -8389.9 | [-18461.9, 1682.0] | -8475.7 | [-18555.6, 1604.2] | | | -8480.9 | [-18561, 1599.1] | |
| Antihypertensives | 1123.6 | [-6744.4, 8991.5] | 1116.1 | [-6780.9, 9013.2] | 1017.7 | [-6847.9, 8883.2] | | | 1116.3 | [-6723.3, 8956.0] | |
| NART error score | -61.32 | [-330.8, 208.2] | -61.61 | [-332.4, 209.1] | -65.8 | [-332.2, 200.6] | | | -62.8 | [-328.4, 202.7] | |
| **Weight parameters** |  |  |  |  |  |  | | |  |  | |
| Childhood | 0.27 | [-0.55, 1.10] | 0.27 | [-0.57, 1.12] | 0.21 | [-0.68, 1.11] | | | 0.22 | [-0.60, 1.05] | |
| Adulthood | 0.72 | [-0.10, 1.55] | 0.72 | [-0.13, 1.57] | 0.78 | [-0.11, 1.68] | | | 0.77 | [-0.05, 1.60] | |
| **Mobility status** |  |  |  |  |  |  | | |  |  | |
| Mobile |  |  | -56.2 | [-5254.6, 5142.2] |  |  | | |  |  | |
| Upward |  |  |  |  | -839.5 | [-6943.5, 5264.4] | | |  |  | |
| Downward |  |  |  |  |  |  | | | 1128.5 | [-6762.1, 8915.1] | |
| AIC | 10826 |  | 10828 |  | 10828 |  | | | 10828 |  | |
| BIC | 10929 |  | 10935 |  | 10935 |  | | | 10935 |  | |
| Degrees of freedom | 25 |  | 26 |  | 26 |  | | | 26 |  | |
| Observations | 464 |  | 464 |  | 464 |  | | | 464 |  | |

Note. The *Constant* denotes average total GMV for a male of average age, average childhood health and average estimated intracranial volume (eTIV). *Socially stable groups*: the class coefficients indicate the class specific deviations from the constant. *Controls*: all models are adjusted for age, sex, eTIV, childhood health, cardiovascular diseases, chronic diseases, blood pressure, waist hip ratio, smoking status, alcohol consumption, physical activity, depression, antidepressants, antihypertensive medications and NART error score. *Weight parameters*: bounded between 0 and 1, they express the relative contribution of childhood SEP and adulthood SEP on total GMV for the socially mobile. A high value of the childhood weight means that childhood class has greater influence on GMV, while a high weight for adulthood means that adulthood class has greater influence on GMV. The mobility (any direction), upward mobility and downward mobility models test the *independent* effect of social mobility on total GMV after accounting for the relative importance of childhood and adulthood SEP. 95% confidence intervals (CI) are given in brackets. AIC: Akaike Information Criteria. BIC: Bayesian Information Criteria. *** p<.001; ** p<.01; * p<.05.

**Table S11.** Point estimates from diagonal reference models (DRMs) estimating social mobility effects on the ventro-medial prefrontal cortex (vmPFC) volume with additional adjustment for lifestyle and disease factors AND performance on the National Adult Reading Test (NART).

|  | ***Baseline model*** |  | ***Mobility***  ***(any direction) model*** |  | ***Upward mobility model*** | | ***Downward mobility model*** | | | |
| --- | --- | --- | --- | --- | --- | --- | --- | --- | --- | --- |
|  | *b* | *95% CI* | *b* | *95% CI* | *b* | *95% CI* | | *b* | | *95% CI* |
| Constant | 10917^***^ | [10055, 11779] | 10925 ^***^ | [10060, 11790] | 10899^***^ | [10037, 11762] | | | 10954 ^***^ | [10091, 11817] |
| **Socially stable groups** |  |  |  |  |  |  | | |  |  |
| Professional / managerial | 108.2 | [-36.2, 252.8] | 105.9 | [-39.8, 251.7] | 121.4 | [-25.7, 268.5] | | | 112.0 | [-32.1, 256.2] |
| Non-manual /skilled manual | -52.4 | [-138.8, 33.8] | -52.4 | [-139.4, 34.4] | -47.5 | [-125.6, 30.5] | | | -44.1 | [-120.5, 32.3] |
| Semi-skilled/unskilled/never worked | -55.7 | [-160.1, 48.5] | -53.4 | [-159.2, 52.3] | -73.8 | [-183.6, 35.8] | | | -67.9 | [-167.1, 31.2] |
| **Controls** |  |  |  |  |  |  | | |  |  |
| Female | -615.1 ^***^ | [-813.1, -417.1] | -613.2 ^***^ | [-811.8, -414.6] | -621.0 ^***^ | [-819.5, -422.6] | | | -609.0 ^***^ | [-806.9, -411.1] |
| Age | -43.7 ^***^ | [-54.8, -32.7] | -43.7 ^***^ | [-54.7, -32.7] | -43.7 ^***^ | [-54.8, -32.7] | | | -43.6 ^***^ | [-54.6, -32.6] |
| eTIV | 0.00^***^ | [0.00, 0.00] | 0.0^***^ | [0.00, 0.00] | 0.0^***^ | [0.00, 0.00] | | | 0.0^***^ | [0.00, 0.00] |
| Childhood health | 5.6 | [-72.4, 83.8] | 6.0 | [-72.1, 84.2] | 5.0 | [-73.0, 83.1] | | | 7.9 | [-70.1, 86.1] |
| Current smoker | -169.8 | [-480.2, 140.5] | -169.0 | [-479.5, 141.3] | -166.5 | [-477.0, 144.0] | | | -157.1 | [-467.8, 153.6] |
| Excessive alcohol | -219.9 | [-471.4, 31.5] | -221.4 | [-473.2, 30.2] | -217.5 | [-468.9, 33.7] | | | -229.4 | [-480.9, 21.9] |
| Waist-Hip ratio | 2.5 | [-3.5, 8.5] | 2.5 | [-3.5, 8.6] | 2.3 | [-3.7, 8.3] | | | 2.5 | [-3.4, 8.5] |
| Chronic diseases | -28.5 | [-175.8, 118.8] | -28.1 | [-175.5, 119.3] | -29.6 | [-176.9, 117.5] | | | -27.0 | [-174.1, 120.0] |
| Cardiovascular diseases | -86.6 | [-305.4, 132.0] | -86.7 | [-305.5, 132.0] | -83.2 | [-301.6, 135.1] | | | -81.4 | [-299.7, 136.7] |
| Systolic blood pressure | 2.3 | [-3.6, 8.3] | 2.3 | [-3.6, 8.3] | 2.2 | [-3.7, 8.2] | | | 2.1 | [-3.8, 8.1] |
| Diastolic blood pressure | -9.7 | [-20.2, 0.78] | -9.7 | [-20.2, 0.78] | -9.4 | [-19.9, 1.00] | | | -9.4 | [-19.9, 1.07] |
| High levels of physical activity | 27.5 | [-158.6, 213.8] | 27.8 | [-158.3, 214.1] | 26.8 | [-159.3, 213.0] | | | 29.0 | [-156.9, 215.0] |
| Depression | -8.3 | [-29.8, 13.1] | -8.5 | [-30.0, 13.0] | -7.5 | [-29.1, 14.0] | | | -8.6 | [-30.1, 12.8] |
| Antidepressants | -116.3 | [-398.7, 165.9] | -116.0 | [-398.4, 166.3] | -117.3 | [-399.4, 164.8] | | | -115.0 | [-396.8, 166.7] |
| Antihypertensives | 22.7 | [-195.3, 240.8] | 21.3 | [-197.0, 239.7] | 23.4 | [-194.4, 241.3] | | | 11.8 | [-206.5, 230.1] |
| NART error score | -3.8 | [-11.0, 3.2] | -3.9 | [-11.1, 3.2] | -3.6 | [-10.8, 3.5] | | | -3.8 | [-10.9, 3.3] |
| **Weight parameters** |  |  |  |  |  |  | | |  |  |
| Childhood | 1.31* | [0.01, 2.60] | 1.30^*^ | [-0.00, 2.61] | 1.53^*^ | [0.01, 3.05] | | | 1.65 | [-0.11, 3.43] |
| Adulthood | -0.31 | [-1.60, 0.98] | -0.30 | [-1.61, 1.00] | -0.53 | [-2.05, 0.98] | | | -0.65 | [-2.43, 1.11] |
| **Mobility status** |  |  |  |  |  |  | | |  |  |
| Mobile |  |  | -18.3 | [-166.0, 129.4] |  |  | | |  |  |
| Upward |  |  |  |  | 92.5 | [-150.4, 335.5] | | |  |  |
| Downward |  |  |  |  |  |  | | | -154.8 | [-408.6, 98.9] |
| AIC | 7509 |  | 7511 |  | 7511 |  | | | 7510 |  |
| BIC | 7613 |  | 7619 |  | 7618 |  | | | 7618 |  |
| Degrees of freedom | 25 |  | 26 |  | 26 |  | | | 26 |  |
| Observations | 464 |  | 464 |  | 464 |  | | | 464 |  |

Note. The *Constant* denotes average vmPFC volume for a male of average age, average childhood health and average estimated intracranial volume (eTIV). *Socially stable groups*: the class coefficients indicate the class specific deviations from the constant. *Controls*: all models are adjusted for age, sex, eTIV, childhood health, cardiovascular diseases, chronic diseases, blood pressure, waist hip ratio, smoking status, alcohol consumption, physical activity, depression, antidepressants, antihypertensive medications and NART error score. *Weight parameters*: bounded between 0 and 1, they express the relative contribution of childhood SEP and adulthood SEP on the vmPFC volume for the socially mobile. A high value of the childhood weight means that childhood class has greater influence on the vmPFC, while a high weight for adulthood means that adulthood class has greater influence on the vmPFC. The mobility (any direction), upward mobility and downward mobility models test the *independent* effect of social mobility on the vmPFC volume after accounting for the relative importance of childhood and adulthood SEP. 95% confidence intervals (CI) are given in brackets. AIC: Akaike Information Criteria. BIC: Bayesian Information Criteria. *** p<.001; ** p<.01; * p<.05.

**Table S12.** Point estimates from diagonal reference models (DRMs) estimating social mobility effects on the anterior cingulate (AC) volume with additional adjustment for lifestyle and disease factors AND performance on the National Adult Reading Test (NART).

|  | ***Baseline model*** |  | ***Mobility***  ***(any direction) model*** |  | ***Upward mobility model*** | | ***Downward mobility model*** | | | |
| --- | --- | --- | --- | --- | --- | --- | --- | --- | --- | --- |
|  | *b* | *95% CI* | *b* | *95% CI* | *b* | *95% CI* | | *b* | | *95% CI* |
| Constant | 7771^***^ | [6602, 9044] | 7790^***^ | [6554, 9025] | 7771^***^ | [6538, 9005] | | | 7850^***^ | [6614, 9086] |
| **Socially stable groups** |  |  |  |  |  |  | | |  |  |
| Professional / managerial | 227.1^*^ | [29.7, 424.6] | 228.8^*^ | [33.9, 423.8] | 210.0^*^ | [-4.8, 424.9] | | | 178.8 | [-37.4, 395.2] |
| Non-manual /skilled manual | 225.8^**^ | [33.6, 418.0] | 220.2^*^ | [26.4, 414.0] | 240.2^**^ | [42.1, 438.4] | | | 248.7^**^ | [57.1, 440.2] |
| Semi-skilled/unskilled/never worked | -453.0^***^ | [-685.4, -220.6] | -449.0^***^ | [-682.04, -216.1] | -450.3^***^ | [-683.5, -217.1] | | | -427.6^***^ | [-663.7, -191.4] |
| **Controls** |  |  |  |  |  |  | | |  |  |
| Female | -88.5 | [-372.2, 195.1] | -82.7 | [-367.2, 201.8] | -92.5 | [-376.6, 191.8] | | | -77.5 | [-361.3, 206.5] |
| Age | -35.0^***^ | [-50.7, -19.2] | -34.9^***^ | [-50.6, -19.1] | -35.3^***^ | [-51.1, -19.5] | | | -35.6^***^ | [-51.3, -19.4] |
| eTIV | 0.00^***^ | [0.00, 0.00] | 0.00^***^ | [0.00, 0.00] | 0.00^***^ | [0.00, 0.00] | | | 0.00^***^ | [0.00, 0.00] |
| Childhood health | -36.9 | [-148.8, 74.9] | -35.8 | [-147.8, 76.1] | -37.1 | [-149.7, 74.1] | | | -33.3 | [-145.2, 78.4] |
| Current smoker | 34.7 | [-414.4, 483.9] | 39.4 | [-409.9, 488.7] | 26.9 | [-423.5, 477.9] | | | 28.3 | [-420.2, 477.2] |
| Excessive alcohol | -396.4^*^ | [-757.0, -35.9] | -400.6^*^ | [-761.4, -39.8] | -395.6^*^ | [-756.1, -35.4] | | | -410.9^*^ | [-715.0, -50.0] |
| Waist-Hip ratio | 1.5 | [-7.2, 10.2] | 1.6 | [-7.0, 10.4] | 1.3 | [-7.3, 10.1] | | | 1.6 | [-7.0, 10.4] |
| Chronic diseases | 207.0^*^ | [-4.9, 419.0] | 207.9^*^ | [-4.0, 420.0] | 206.0^*^ | [-5.7, 417.1] | | | 206.8^*^ | [-4.3, 417.2] |
| Cardiovascular diseases | 147.8 | [-167.5, 463.1] | 147.7 | [-167.5, 462.9] | 146.7 | [-168.7, 462.2] | | | 143.3 | [-171.8, 458.0] |
| Systolic blood pressure | 3.3 | [-5.2, 11.9] | 3.3 | [-5.2, 11.9] | 3.2 | [-5.4, 11.8] | | | 3.0 | [-5.5, 11.6] |
| Diastolic blood pressure | -4.2 | [-19.2, 10.8] | -4.3 | [-19.4, 10.8] | -4.0 | [-19.3, 11.0] | | | -4.1 | [-19.2, 10.9] |
| High levels of physical activity | -22.8 | [-289.1, 243.4] | -22.6 | [-288.9, 243.5] | -20.5 | [-286.5, 245.9] | | | -14.3 | [-392.0, 82.6] |
| Depression | -2.5 | [-33.4, 28.3] | -3.0 | [-34.0, 27.8] | -2.0 | [-32.9, 28.7] | | | -3.0 | [-33.8, 27.7] |
| Antidepressants | -138.7 | [-542.6, 265.0] | -137.9 | [-541.7, 265.8] | -137.3 | [-540.3, 266.2] | | | -129.6 | [-532.5, 273.2] |
| Antihypertensives | -129.9 | [-442.0, 182.1] | -135.1 | [-447.8, 177.5] | -124.2 | [-437, 188.5] | | | -131.6 | [-443.2, 180.0] |
| NART error score | 3.0 | [-7.2, 13.4] | 3.0 | [-7.2, 13.4] | 2.8 | [-7.5, 13.3] | | | 2.35 | [-8.03, 12.7] |
| **Weight parameters** |  |  |  |  |  |  | | |  |  |
| Childhood | 0.27 | [-0.01, 0.55] | 0.25 | [-0.04, 0.55] | 0.33 | [-0.03, 0.70] | | | 0.40^*^ | [0.07, 0.73] |
| Adulthood | 0.72^***^ | [0.44, 1.01] | 0.75^***^ | [0.44, 1.04] | 0.66^***^ | [0.29, 1.03] | | | 0.59^***^ | [0.26, 0.92] |
| **Mobility status** |  |  |  |  |  |  | | |  |  |
| Mobile |  |  | -54.2 | [-263.6, 155.2] |  |  | | |  |  |
| Upward |  |  |  |  | 66.6 | [-214.4, 348.1] | | |  |  |
| Downward |  |  |  |  |  |  | | | -222.6 | [-538.4, 93.1] |
| AIC | 7842 |  | 7844 |  | 7844 |  | | | 7843 |  |
| BIC | 7946 |  | 7952 |  | 7952 |  | | | 7950 |  |
| Degrees of freedom | 25 |  | 26 |  | 26 |  | | | 26 |  |
| Observations | 464 |  | 464 |  | 464 |  | | | 464 |  |

Note. The *Constant* denotes average AC volume for a male of average age, average childhood health and average estimated intracranial volume (eTIV). *Socially stable groups*: the class coefficients indicate the class specific deviations from the constant. *Controls*: all models are adjusted for age, sex, eTIV, childhood health, cardiovascular diseases, chronic diseases, blood pressure, waist hip ratio, smoking status, alcohol consumption, physical activity, depression, antidepressants, antihypertensive medications and NART error score. *Weight parameters*: bounded between 0 and 1, they express the relative contribution of childhood SEP and adulthood SEP on the AC volume for the socially mobile. A high value of the childhood weight means that childhood class has greater influence on the AC, while a high weight for adulthood means that adulthood class has greater influence on the AC. The mobility (any direction), upward mobility and downward mobility models test the *independent* effect of social mobility on the AC volume after accounting for the relative importance of childhood and adulthood SEP. 95% confidence intervals (CI) are given in brackets. AIC: Akaike Information Criteria. BIC: Bayesian Information Criteria. *** p<.001; ** p<.01; * p<.05.

**Table S13**. The effect of social mobility on the Montreal Cognitive Assessment (MoCA) and the Mini-Mental State Examination (MMSE) using binary logistic regression.

|  | **MOCA** |  | **MMSE** |  |
| --- | --- | --- | --- | --- |
|  | **Odds Ratio** | **95% CI** | **Odds Ratio** | **95% CI** |
| Constant | 0.00^***^ | 0.00, 0.01 | 0.00^***^ | 0.00, 0.01 |
| **Mobility status groups** |  |  |  |  |
| Stable professional/ managerial | [REF] | [REF] | [REF] | [REF] |
| Downwardly mobile | 6.36^***^ | 3.10, 13.04 | 9.42^***^ | 3.73, 23.77 |
| Stable non-manual/ skilled manual | 4.46^***^ | 2.17, 9.17 | 5.70^***^ | 2.24, 14.48 |
| Stable semi-skilled/ unskilled/ never worked | 12.39^***^ | 6.02, 25.50 | 12.29^***^ | 4.82, 31.32 |
| Upwardly mobile | 3.52^***^ | 1.74, 7.12 | 3.77^**^ | 1.49, 9.51 |
| **Controls** |  |  |  |  |
| Age | 1.12^***^ | 1.11, 1.14 | 1.09^***^ | 1.08, 1.11 |
| Sex | 1.01 | 0.79, 1.29 | 0.85 | 0.65, 1.13 |
| Childhood health | 1.11 | 0.98, 1.26 | 1.35^***^ | 1.19, 1.53 |

Note. Adjusted for age, sex and childhood health (mean centered). 95% CI = Confidence Intervals.

*** p<.001; ** p<.01; * p<.05.

**Table S14**. The effect of social mobility on total grey matter volume (GMV), ventromedial prefrontal cortex (vmPFC) volume and anterior cingulate (AC) volume using ordinary least squares regression.

|  | **GMV** |  | **vmPFC** |  | **AC** |  |
| --- | --- | --- | --- | --- | --- | --- |
|  | **Coef.** | **95% CI** | **Coef.** | **95% CI** | **Coef.** | **95% CI** |
| Constant | 597317^***^ | 590322, 604313 | 10650^***^ | 10457, 10843 | 8249^***^ | 7971, 8526 |
| **Mobility status groups** |  |  |  |  |  |  |
| Stable professional/ managerial | [REF] | [REF] | [REF] | [REF] | [REF] | [REF] |
| Downwardly mobile | -3896 | -12795, 5001 | -171.5 | -417.1, 74.1 | -334.3^*^ | -687, 18.6 |
| Stable non-manual/ skilled manual | -1393 | -10270, 7483 | -175.4 | -420.4, 69.6 | 18.0 | -334.0, 370.0 |
| Stable semi-skilled/ unskilled/ never worked | -13997^**^ | -24793, -3202 | -383.9^*^ | -681.9, -86.1 | -532.1^**^ | -960.2, -104.0 |
| Upwardly mobile | -2484 | -9974, 5005 | -208.9^*^ | -415.6, -2.2 | -41.3 | -338.3, 255.7 |
| **Controls** |  |  |  |  |  |  |
| Age | -2815^***^ | -3175, -2456 | -42.2^***^ | -52.1, -32.2 | -32.7^***^ | -46.9, -18.4 |
| Sex | -18309^***^ | -24726, -11891 | -603.7^***^ | -780.8, -426.6 | -9.7 | -264.2, 244.8 |
| Childhood health | -1165 | -3990, 1660 | -10.3 | -88.3, 67.6 | -40.3 | -152.3, 71.7 |
| Estimated intracranial volume | 0.16^***^ | 0.14, 0.18 | 0.0^***^ | 0.00, 0.00 | 0.0^***^ | 0.00, 0.00 |

Note. Adjusted for age, sex, childhood health and estimated intracranial volume (mean centered). 95% CI = Confidence Intervals.

*** p<.001; ** p<.01; * p<.05.
